# Differential effects of intense exercise and pollution on the airways in a murine model

**DOI:** 10.1186/s12989-021-00401-6

**Published:** 2021-03-15

**Authors:** Tatjana Decaesteker, Eliane Vanhoffelen, Kristel Trekels, Anne-Charlotte Jonckheere, Jonathan Cremer, Arno Vanstapel, Ellen Dilissen, Dominique Bullens, Lieven J. Dupont, Jeroen A. Vanoirbeek

**Affiliations:** 1 Laboratory of Respiratory Diseases and Thoracic Surgery (BREATHE), Department of Chronic Diseases and Metabolism (CHROMETA), KU Leuven, University of Leuven, Herestraat 49, mailbox 706, 3000 Leuven, Belgium; 2 Centre for Environment and Health, Department of Public Health and Primary Care, KU Leuven, Leuven, Belgium; 3 Allergy and Clinical Immunology Research Group, Department of Microbiology, Immunology and Transplantation, KU Leuven, Leuven, Belgium; 4 Department of Paediatrics, University Hospitals Leuven, Leuven, Belgium; 5 Department of Respiratory Diseases, University Hospitals Leuven, KU Leuven, Leuven, Belgium

**Keywords:** Diesel exhaust particles, Exercise-induced bronchoconstriction, Tight junctions, Dendritic cells, Non-allergic

## Abstract

**Background:**

Exercise-induced bronchoconstriction (EIB) is a transient airway narrowing, occurring during or shortly after intensive exercise. It is highly prevalent in non-asthmatic outdoor endurance athletes suggesting an important contribution of air pollution in the development of EIB. Therefore, more research is necessary to investigate the combination of exercise and pollutants on the airways.

**Methods:**

Balbc/ByJ mice were intranasally challenged 5 days a week for 3 weeks with saline or 0.2 mg/ml diesel exhaust particles (DEP), prior to a daily incremental running session or non-exercise session. Once a week, the early ventilatory response was measured and lung function was determined at day 24. Airway inflammation and cytokine levels were evaluated in bronchoalveolar lavage fluid. Furthermore, innate lymphoid cells, dendritic cells and tight junction mRNA expression were determined in lung tissue.

**Results:**

Submaximal exercise resulted in acute alterations of the breathing pattern and significantly improved FEV_0.1_ at day 24. DEP exposure induced neutrophilic airway inflammation, accompanied with increased percentages of CD11b^+^ DC in lung tissue and pro-inflammatory cytokines, such as IL-13, MCP-1, GM-CSF and KC. Occludin and claudin-1(Cldn-1) expression were respectively increased and decreased by DEP exposure. Whereas, exercise increased Cldn-3 and Cldn-18 expression. Combining exercise and DEP exposure resulted in significantly increased SP-D levels in the airways.

**Conclusion:**

DEP exposure induced typical airway neutrophilia, DC recruitment and pro-inflammatory cytokine production. Whereas, intensive exercise induced changes of the breathing pattern. The combination of both triggers resulted in a dysregulation of tight junction expression, suggesting that intensive exercise in polluted environments can induce important changes in the airway physiology and integrity.

**Supplementary Information:**

The online version contains supplementary material available at 10.1186/s12989-021-00401-6.

## Background

Exercise-induced respiratory symptoms are highly prevalent in elite athletes, who are mostly not diagnosed with an underlying asthma pathology or without a familial asthma history [1]. Epidemiological studies have shown differences in the presence of exercise-induced respiratory symptoms between distinct sport disciplines, with the highest prevalence in swimming, endurance sports and winter sports, which suggests the importance of the specific environmental conditions [1–4]. During exercise, ventilation increases up to 150 L/min in healthy adults and even beyond 200 L/min in elite athletes, resulting in an increased inhalation of potential harmful environmental triggers [5, 6].

Several underlying mechanisms are proposed to explain the development of exercise-induced respiratory symptoms in athletes [1, 7]. Airway dehydration and cooling (“The osmotic and thermal hypothesis”) were considered as major causes of EIB [1, 7, 8]. However, recent evidence suggests the involvement of other mechanistic pathways, such as epithelial injury [9, 10]. Increased levels of epithelial bronchial cells and epithelial damage markers, such as Club Cell protein-16 (CC16), were found in sputum of marathon runners post-exercise [9]. Similar inflammatory responses were also described in elite swimmers; in whom sputum uric acid, high mobility group box-1 and serum CC16 were elevated after 90-min intensive swimming [10]. These epithelial, cellular and biochemical changes were mostly accompanied with increased neutrophilic airway inflammation [1, 5, 9, 10]. Currently it is hypothesised that epithelial injury is a consequence of increased shear stress due to a high ventilation and/or environmental exposures.

Nowadays, air pollution is a major issue and the negative health effects of pollutants have been shown repeatedly [11–13]. This is also true for elite athletes, who perform intensive outdoor activities [14–16]. The positive effects of moderate-intensity exercise on health are widely recognised, resulting in a massive increase of running events for non-elite athletes. Consequently, the general population is performing more high-intensity outdoor exercise, often in conditions with (high) air pollution. This might lead to an increased risk of cardiopulmonary problems and immune alterations due to exercise in polluted areas, as shown in a few studies [17].

It has been extensively proven that polluting compounds, such as particulate matter (PM), diesel exhaust particles (DEP), nitric oxide (NO) and O_3_, induce inflammatory and immunological effects in both humans and murine models [17–19]. PM exposure mostly enhances a mixed Th2/Th17 immune response, associated with airway hyperresponsiveness, neutrophilic airway inflammation and airway cytokine release (IL-5, IL-13, IFN-γ and IL-17A) [20, 21]. Estrella et al. summarized that DEP/PM is also able to inhibit the proliferation of ILC1 and NK cells and to stimulate ILC2 [19].

Murine models have mainly focused on the anti-inflammatory effects of aerobic exercise on allergic diseases and not on the (pro-inflammatory) effects of more intensive exercise [22, 23]. Furthermore, it is not known if air pollution inhibits or enhances the pro- and/or anti-inflammatory effects of intensive exercise. In our study, we developed a submaximal 3-week running model to study the combined effect of intensive exercise and DEP exposure on airway inflammation, hyperresponsiveness, airway integrity and airway immune responses in mice.

## Methods

### Reagents

Diesel particulate matter (diesel exhaust particles; DEP) (NIST2975, CAS: 1333-86-4), characterised by the National Institute of Standards and Technology (NIST), and Tween®20 (CAS:9005-64-5) were obtained from Sigma-Aldrich (Bornem, Belgium). The vehicle saline + 0.2% Tween®20 is used to dissolve the diesel exhaust particles. An instillation concentration of 0.2 mg/ml was used, which mimics the real-life human exposure to diesel exhaust particles during outdoor physical activity in Flanders. Calculations are described in detail in the discussion section. Pentobarbital (Dolethal®) was obtained from the internal animal facility at the University of Leuven.

### Mice

Juvenile male Balb/cByJ mice (7–8 weeks old) were obtained from Charles River Laboratories, Belgium. All mice were housed in a conventional animal facility with 12 h dark/light cycles. They were housed in individually ventilated cages and received water and food ad libitum. Mice were daily weighted, shown in Appendix A Figure S1. The study was approved by the local Ethical Committee for animal experiments of KU Leuven, Leuven, Belgium (P065/2017).

### Exercise and instillation protocol

**Fig. 1:**
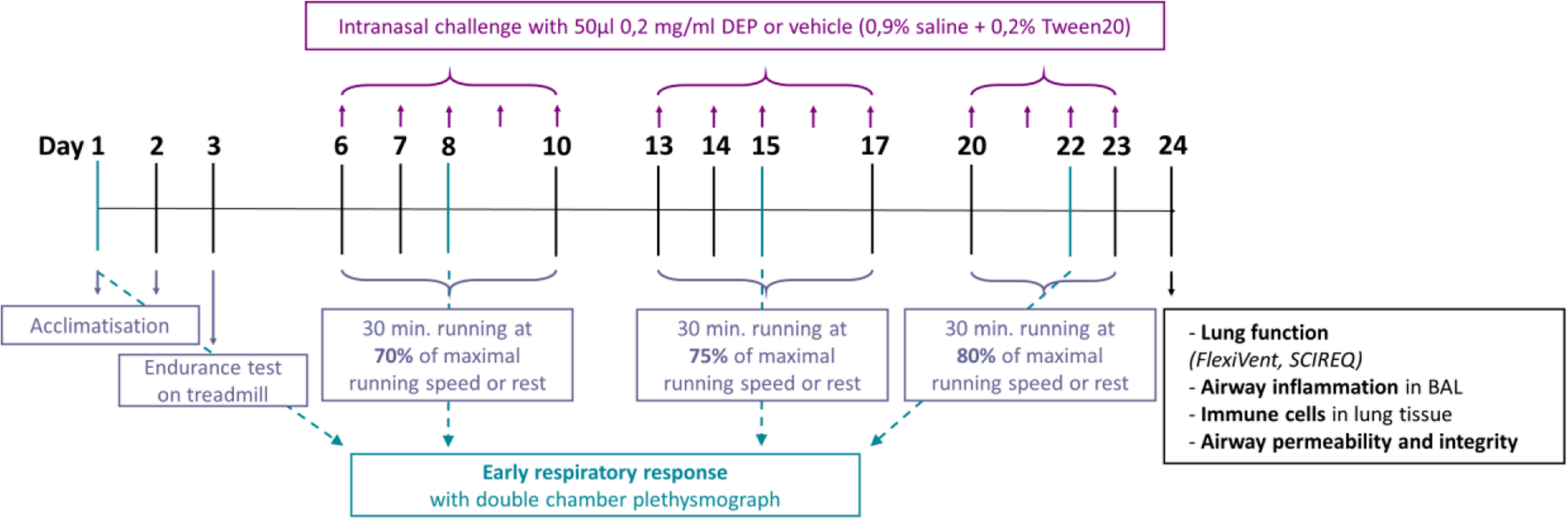
Study design used in experiment 1 and 2

On days 1 and 2, all mice ran for 15 min at a low speed to acclimatize to the system and exercise, consisting of 5 min running without movement of the treadmill and 10 min at 6 m/min. On day 3, the mice performed an endurance test to determine their maximal running capacity. The mice started running at 3 m/min for 5 min, followed by an increase of 1 m/min until exhaustion, which was determined as the moment that the mice were not able to follow the speed of the treadmill. From day 6, mice ran for 30 min during 5 consecutive days per week, for 3 weeks. The speed of the treadmill was set at 6 m/min for 5 min, followed by a 1 m/min increase until 70% (week 1), 75% (week 2) and 80% (week 3) of their individual maximum speed was reached. Thirty minutes prior to the daily running session, mice were intranasally instilled with 50 μl 0.2 mg/ml DEP, dissolved in the vehicle (saline + 0.2% Tween20), or with the vehicle itself. If mice stopped running during the protocol, they were stimulated with a spoon to continue running. We included two groups of mice that did not run and only received intranasal instillation with saline (vehicle + non-exercise is indicated as Sal/NE) or DEP (DEP + non-exercise is indicated as DEP/NE) and two groups of mice that performed the running experiments and either got challenged with saline (vehicle + exercise is indicated as Sal/E) or with DEP (DEP + exercise is indicated as DEP/E). The study design is shown in Fig. 1 and a detailed running scheme is described in Appendix A Table S1.

### Endpoints

We performed the experiment in three separate cohorts with randomization of the mice. In a first cohort (8–9 mice per group), we evaluated the early ventilatory response, airway hyperreactivity, airway inflammation, DEP-uptake and airway integrity. We measured cytokine levels in bronchoalveolar lavage, tight junction expression in lung tissue and evaluated airway permeability and airway histology. In a second cohort (12 mice per group), we determined subpopulations of dendritic cells and innate lymphoid cells. Bronchoalveolar lavages (BAL) were performed to confirm the airway inflammation and cytokine levels in BAL. In a third cohort (8 mice per group), the mice followed the above described exposure and running protocol, which was followed by a resting period of 3 weeks, without exercise and intranasal instillations to evaluate the post-exposure repair. After 3 weeks exposure (with or without exercise) and 3 weeks without exposure and exercise, mice were sacrificed to determine airway hyperreactivity, airway inflammation, DEP-uptake and airway cytokine levels (experimental set-up shown in Appendix A Fig. S2).

As each mouse followed their individual running scheme, based on their maximal running capacity, an individual running distance was calculated for each mouse, reflecting differences in running intensity (shown in Appendix A Table S2). These total distances were used to evaluate the correlation between running intensity and several measured parameters.

### Airway measurements

#### Early ventilatory response

Once a week (on days 1, 8, 15 and 22), the respiratory pattern was recorded and parameters as peak inspiratory volume (PIF), peak expiratory volume (PEF), minute volume (MV), tidal volume (TV), breathing frequency (f), inspiratory time (Ti) and expiratory time (Te) were assessed in a non-invasive way before and immediately after exercise using double chamber plethysmography (DCP, Emka Technologies, Paris, France). A detailed protocol is available in Appendix B.

#### Airway hyperreactivity

Twenty-four hours after the last running session, airway hyperreactivity (AHR) to increasing concentrations methacholine (0, 1.25, 2.5, 5, 10, 20 mg/ml) and lung function parameters, such as airway resistance, forced expiratory volume in 0.1 s (FEV_0.1_), forced vital capacity (FVC), peak expiratory flow (PEF) and Tiffeneau-index (FEV_0.1_/FVC), were measured using the forced oscillation and negative pressure forced expiration technique, respectively (FlexiVent 7.3, SCIREQ, Montreal, Canada). Mice were anesthetized with pentobarbital (120 mg/kg body weight, Dolethal®). A detailed protocol is available in Appendix B.

### Serum analysis

After measuring airway hyperreactivity, mice were sacrificed. Blood was sampled from the retro-orbital plexus, centrifuged (14,000 g, 4 °C, 10 min) and serum samples were stored at − 80 °C until analysis. Serum surfactant protein D (SP-D) was measured by the use of ELISA (Mouse SP-D DuoSet ELISA (DY6839-05), R&D Systems, Minneapolis, US) and serum uric acid (UA) by the use of an Amplex Red Uric Acid/Uricase Assay kit (ThermoFisher Scientific, Massachusetts, US), both according to manufacturers’ guidelines. Detection limits were 62.5 μg/mL and 100 nM, respectively. Samples were undiluted for measurement.

### Lung analysis

#### Broncho-alveolar lavage (BAL)

The lungs were lavaged three times in situ with 0.7 ml sterile saline (0.9% NaCl), and the recovered fluid was pooled. Cells were counted using a Bürker hemocytometer (total cell count) and the BAL fluid was centrifuged (1000 g, 10 min). For differential cell count, 250 μl of the resuspended cells (100,000 cells/ml) were spun (300 g, 6 min) (Cytospin 3, Shandon, TechGen, Zellik, Belgium) onto microscope slides, air-dried and stained (Diff-Quik® method, ThermoFisher Scientific, Massachusetts, US). For each sample, 200 cells were counted for the enumeration of macrophages, eosinophils, neutrophils and lymphocytes.

#### DEP-uptake analysis

To evaluate the DEP-uptake by the macrophages, cytospin slides of BAL were examined. Firstly, the percentage of loaded macrophages was determined by manually counting a total of 100 macrophages. Secondly, the number of diesel exhaust particles was determined by the use of ImageJ software (NIH, Maryland, US), according the method described by Bai et al. [24]. Cells were manually delineated and the software automatically counted the number diesel exhaust particles in the indicated area. Fifty macrophages per mice were analysed and the average was calculated.

#### Bradford assay

Total protein level was measured in BAL fluid using a Bradford Assay (Bio-Rad, München, Germany). One hundred sixty microliters sample, 1/5 dilution in distilled water, and standard were added to the plate, followed by 40 μl Dye Reagent to elicit a colour reaction. Absorbance was measured at 595 nm. Following standard curve was used: 31.25 μg/ml, 15.625 μg/ml, 6.25 μg/ml, 3.125 μg/ml, 1.56 μg/ml, 0 μg/ml. Detection limit was 1.25 μg/ml.

#### Cytokine analysis

Cytokine and chemokine concentrations of interferon-γ (IFN-γ), interleukin-1β (IL-1β), IL-6, keratinocyte-derived chemokine (KC), tumor necrosis factor-α (TNF-α), granulocyte-macrophage colony-stimulating factor (GM-CSF), IL-13, IL-17A, IL-17F, IL-25, IL-33, macrophage inflammatory protein-2 (MIP-2) and monocyte chemoattractant protein-1 (MCP-1) were measured in BAL fluid using a U-plex Assay (Meso Scale Diagnostics, Maryland, USA). Measurements were performed according manufacturers’ instructions. Detection limits were 0.27 pg/ml, 1.15 pg/ml, 8.40 pg/ml, 0.64 pg/ml, 0.38 pg/ml, 0.23 pg/ml, 16.60 pg/ml, 0.14 pg/ml, 14.70 pg/ml, 1.04 pg/ml, 0.23 pg/ml, 0.44 pg/ml and 2.48 pg/ml respectively.

#### Tight junction mRNA expression (RT-qPCR)

During autopsy, the left lung was collected, immediately snap frozen and stored at − 80 °C. The snap frozen lungs were homogenized for RNA extraction in RLT RNA lysis buffer (Qiagen, Hilden, Germany) with lysing matrix D bead using the FastPrep-24 (MP Biomedicals, Irvine, USA). Afterwards, homogenates were centrifuged at room temperature (full speed, 3 min, twice).

RNA from the lung homogenates was isolated using the Qiagen Mini RNeasy Kit (Qiagen, Hilden, Germany), according manufacturers’ instructions. Concentration and quality were checked with the NanoDrop One (ThermoFisher Scientific, Massachusetts, US). Five μg RNA was converted into cDNA with the High-Capacity cDNA Reverse Transcription kit with RNase inhibitor (Applied Biosystems™, Californië, US) and used to determine gene expression.

Gene expression for zona occludens-1, occludin, claudin-1, claudin-3, claudin-4, claudin-18 and reference genes, β-actin (bACT) and ribosomal protein L13a (RPL13a) were evaluated via RT-qPCR (detailed protocol in Appendix B). cDNA plasmid standards, consisting of purified plasmid DNA specific for each target, were used to quantify the amount of target gene in the unknown samples, as previously described [25]. Primer sequences can be found in Appendix B.

#### Histology

Lungs were instilled with 4% formaldehyde until full inflation of all lobes. An experienced pathologist evaluated lung injury, airway inflammation and DEP deposition in the airways on slides of paraffin sections (5 μm thickness) stained with haemalum and eosin, in a blinded manner. Histology was performed of the collected lungs of experiment 1.

#### Single cell suspension

Lung tissue was collected during autopsy and directly processed to prepare a single cell suspension. This was obtained using digestion medium (RPMI 1640 supplemented with 5% FCS, 2 mM L-glutamine, 0.05 mM 2-mercaptoethanol [Gibco, Invitrogen, Paisley, United Kingdom], 100 U/ml penicillin, 100 mg/ml streptomycin [Invitrogen], 1 mg/ml collagenase type 2 [Worthington Biochemical, Lakewood, NY], and 0.02 mg/ml DNase I [grade II from bovine pancreas, Boehringer Ingelheim, Ingelheim, Germany]) for 45 min at 37 °C and 5% CO_2_. Red blood cells were lysed using ammonium chloride potassium lysing buffer. Cells were counted using a Bürker hemocytometer and resuspended (10^7^ cells/ml) in PBS.

#### Dendritic cell subpopulations

To minimize nonspecific binding, 2 million cells of lung suspension, were pre-incubated with an anti-CD16/CD32 antibody (Clone 2.4G2, BD Biosciences, Erembodegem, Belgium). After viability staining, using the Zombie Aqua™ Fixable Viability Kit, cells were labelled with combinations of anti-mouse fluorochrome-conjugated mAbs against CD45 (clone 30-F11), CD11c (clone N418), MHCII (clone M5/114.15.2), CD11b (clone M1/70), CD103 (clone 2E7), CD64 (clone X54-5/7.1) and Siglec-H (clone 551) (all from Biolegend, San Diego, California). Sample acquisition was performed on a LSR Fortessa SORP flow cytometer running DIVA software (BD Biosciences, Erembodegem, Belgium). FlowJo software (BD Bioscience, Ashland, Oregon) was used for data analysis. Gating strategy is available in Appendix A Fig. S3. Flow cytometry configuration are available in Appendix B.

#### Innate lymphoid cell subpopulations

Four million cells were stained with Fixable Viability Dye FluorTM 780 (eBioscience, Invitrogen, Merelbeke, Belgium) to perform a viability staining and pre-incubated with CD16/CD32 (clone 2.4G2, BD Biosciences, Erembodegem, Belgium) to block non-antigen-specific binding of immunoglobulins. Combinations of anti-mouse fluorochrome-conjugated mAbs against CD45 (clone 30-F11), CD127 (clone SB/199), CD90.2 (clone 53-2.1), KLRG-1 (clone 2F1), CD11b (clone M1/70), CD19 (clone 1D3), CD3e (clone G4.18), CD45RB (clone 16A), CD49b (Clone AK-7), CD5 (clone 53-7.3), TCRγδ (clone GL3), Ter-119 (clone TER-119) (all from BD Biosciences, Erembodegem, Belgium), RORγT (clone B2D)(ThermoFisher Scientific, Massachusetts, US), NKp46 (clone 29A1.4), Ly6G (Clone 1A8) and CD94 (clone 20d5)(all from Biolegend, San Diego, California) were used to label the cells. Sample acquisition was performed on a LSR Fortessa SORP flow cytometer running DIVA software (BD Biosciences, Erembodegem, Belgium). Data analysis was performed by using FlowJo software. Gating strategy available in Appendix A Fig. S4. Flow cytometry configuration are available in Appendix B.

### Data analysis

The data are presented as means with standard deviation (SD), or as individual mice and group mean. Normality of distribution was assessed by the Kolmogorov-Smirnov test, followed by a One-Way parametric ANOVA combined with a Bonferroni multiple comparison post hoc test to evaluate intergroup differences. To evaluate combination effects a Two-Way ANOVA combined with a Bonferroni multiple comparison post hoc test was used. (Graph Pad Prism 8.02. Graphpad Software Inc., San Diego, USA). A level of *p* < 0.05 (two tailed) was considered significant.

## Results

### Early ventilatory response

#### Longitudinal changes of the pre-exposure breathing pattern

**Fig. 2:**
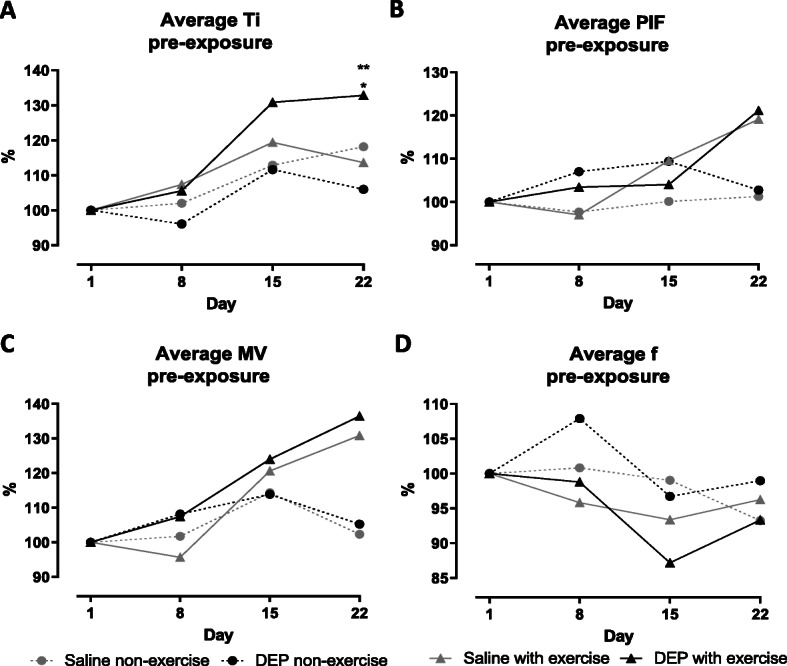
Longitudinal changes of pre-exposure breathing pattern at day 1, 8, 15 and 22. Breathing parameters were measured on days 1, 8, 15 and 22 prior to the exposure and running or rest session using a double chamber plethysmograph. **a** The average inspiratory time (Ti) pre-exposure. **b** The average peak inspiratory flow (PIF) pre-exposure. **c** The average minute volume (MV) pre-exposure. **d** The average breathing frequency (f) pre-exposure. Data is shown as mean (*n* = 7–9 mice per group). *p < 0.05 for saline exposed exercised mice compared with DEP exposed exercised mice, **p < 0.01 for DEP exposed non-exercised mice compared with DEP exposed exercised mice (Two-Way ANOVA)

Figure 2a shows a significant higher Ti in DEP/E mice compared to the other groups at day 22 (*p* < 0.01). Breathing frequency (f) varied over time but did not show significant changes, shown in Fig. 2d). Te, PEF, EV and TV were not significantly changed and are shown in Appendix A (Fig. S5).

#### Exposure-induced changes of the breathing pattern

**Fig. 3:**
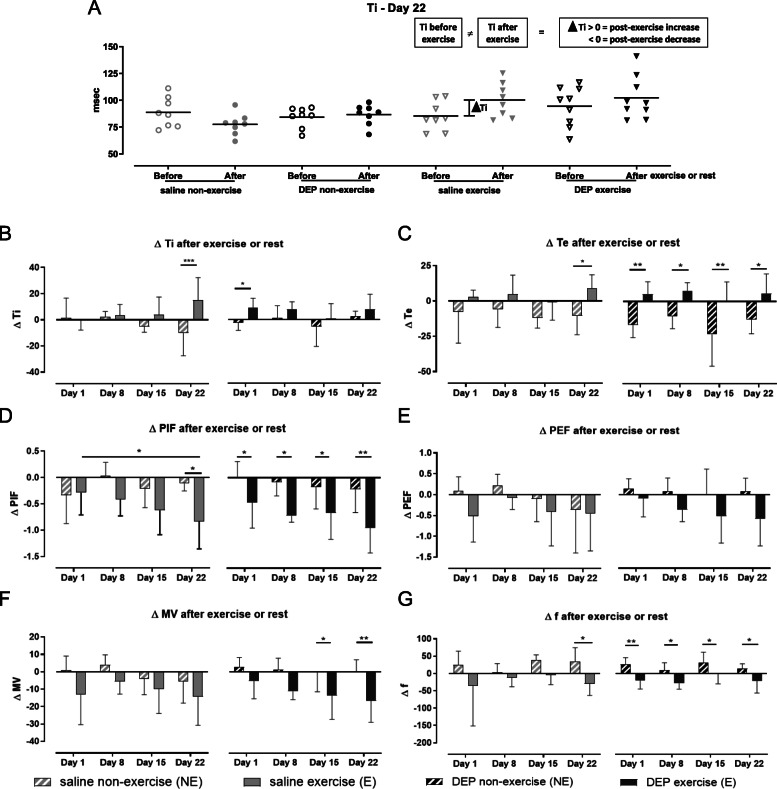
Exposure-induced changes of the breathing pattern. Breathing parameters were measured on days 1, 8, 15 and 22 prior to and immediately after the exposure and running or rest session using a double chamber plethysmograph. The exposure-induced changes were determined by calculating the difference between the measured values pre- and post-exposure. If the difference > 0, an exercise-induced increase is identified. If the difference < 0, an exercise-induced decrease was identified, as shown in (**a)** for the inspiratory time at day 22. **b** The average exposure-induced changes of the inspiratory time (ΔTi). **c** The average exposure-induced changes of the expiratory time (ΔTe). **d** The average exposure-induced changes of the peak inspiratory flow (ΔPIF). **e** The average exposure-induced changes of the peak expiratory flow (ΔPEF). **f** The average exposure-induced changes of the minute volume (ΔMV). **g** The average exposure-induced changes of the breathing frequency (Δf). Data are shown as mean with SD (*n* = 7–8 mice/group). *p < 0.05, **p < 0.01, ***p < 0.001 (Two-Way ANOVA)

On days 1, 8, 15 and 22, we have measured the breathing pattern both before and after exercise, which allows us to calculate the differences between both measurements for each individual mouse. An example of the individual pre- and post-exercise or non-exercise data with the corresponding calculation of the difference is shown for Ti at day 22 in Fig. 3a.

Ti and Te were significantly increased in both exercise groups after the running session compared with the non-exercise groups at day 22 for Ti and Te of Sal/E mice (*p* < 0.001 for Ti and *p* < 0.05 for Te), at day 1 for Ti of DEP/E mice (*p* < 0.05), at day 22 for Te of Sal/E mice (*p* < 0.05) and at all time points for Te of DEP/E mice compared to the non-exercised control group (*p* < 0.05 at day 8 and 15, *p* < 0.01 at day 1 and 22) (shown in Fig. 3b and c). PIF, PEF, MV and f were decreased after the running protocol in both the DEP and saline exposed groups, while non-exercised mice did not show changes of PIF, PEF, MV and f after the rest period. A significant decrease of PIF post-exercise was seen at day 22 for the Sal/E mice (*p* < 0.05) and at all time points for DEP exposed mice compared to their non-exercised counterparts (*p* < 0.05 at day 1, 8 and 15, *p* < 0.01 at day 22), while the decreased PEF in exercised mice did not reach significance (Fig. 3d and e, respectively). Figure 3f shows a significant decrease of MV in exercised mice but was only significantly different from their non-exercise counterparts at days 15 and 22 for DEP exposed mice (*p* < 0.05 at day 15 and *p* < 0.01 at day 22). The breathing frequency, shown in Fig. 3g, was slightly decreased post-exposure in exercised mice, while non-exercised mice showed a slight increase of post-exposure, resulting in significant differences at day 22 for the saline exposed mice (*p* < 0.05) and at all time points for DEP exposed mice (*p* < 0.05 at day 8, 15 and 22, *p* < 0.01 at day 1).

### Lung function

#### Lung function at day 24

**Fig. 4:**
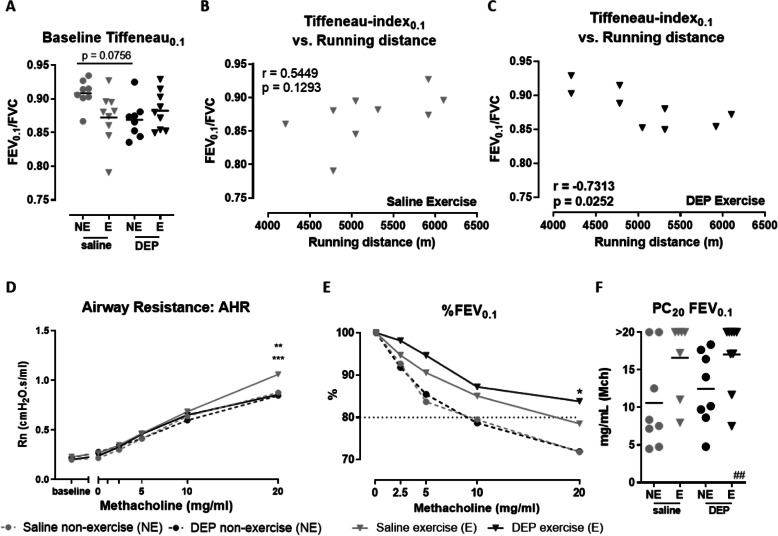
Baseline lung function and airway hyperreactivity on day 24. Lung function parameters and airway hyperreactivity were assessed at day 24, 24 h after the last exposure and running or rest session, using the FlexiVent system. **a** Baseline Tiffeneau-index_0.1_ was calculated as FEV_0.1_/FVC. (*p* = 0.0756, Two-Way ANOVA). **b** Correlation between baseline Tiffeneau-index_0.1_ and running distance of saline exposed exercised mice (*r* = 0.5449, *p* = 0.1293, Pearson correlation). **c** Correlation between baseline Tiffeneau-index_0.1_ and running distance of DEP exposed exercised mice (*r* = − 0.7313, *p* = 0.0252, Pearson correlation). **d** The group average dose-response of the airway resistance (Rn) to methacholine (0–20 mg/ml) was measured using a forced oscillation technique (QP3). **p < 0.01 for Sal/NE compared with Sal/E, ***p < 0.001 for Sal/E compared with DEP/E (Two-Way ANOVA). **e** The group average dose-response of FEV_0.1_ (%) to methacholine (0–20 mg/ml) was measured using the negative pressure forced expiration (NPFE) manoeuvre. *p < 0.05 DEP/NE compared with DEP/E (Two-Way ANOVA). **f** The provocative methacholine concentration inducing a 20% decrease in FEV_0.1_, relative to the baseline FEV_0.1_, was calculated based on the dose-response curve of FEV_0.1_ (%), shown in (**e**). (exercise-effect with ^##^*p* = 0.0080, Two-Way ANOVA). Individual data point in figure **a** and **f** are shown with mean (*n* = 7–9 mice per group)

None of the baseline lung function parameters (Rn, FVC, FEV_0.1_, FEV_0.2_, PEF) showed significant differences compared to each other (data not shown). Baseline FVC and FEV_0.1_, were used to calculate the Tiffeneau-index_0.1_ (FEV_0.1_/FVC ratio reflecting airway obstruction), which was also not statistically different (Fig. 4a).

Tiffeneau-index_0.1_ was positively correlated with running distance in Sal/E mice (*r* = 0.5449, *p* = 0.1293, Pearson correlation) (Fig. 4b), while there was a significant negative correlation in DEP/E mice (*r* = − 0.7313, *p* = 0.0252, Pearson correlation) (Fig. 4c).

#### Airway hyperreactivity at day 24

Figure 4d-e show the dose-response of methacholine-induced airway hyperreactivity. Airway hyperreactivity (Rn) was significantly higher in Sal/E mice compared to the other experimental groups (*p* < 0.01 compared to Sal/NE mice and *p* < 0.001 compared to DEP/E and DEP/NE mice (Fig. 4d)).

FEV_0.1_ (%) was also significantly lower in non-exercised mice compared to exercised mice, at 20 mg/ml when comparing DEP/E mice with the Sal/NE and DEP/NE mice (*p* < 0.05, Fig. 4e). This was also reflected in the PC_20_, calculated based on the FEV_0.1_ (%), showing a significant exercise effect on PC_20_ (Two-way ANOVA: *p* = 0.0080, Fig. 4f).

### Lung inflammatory response

**Fig. 5:**
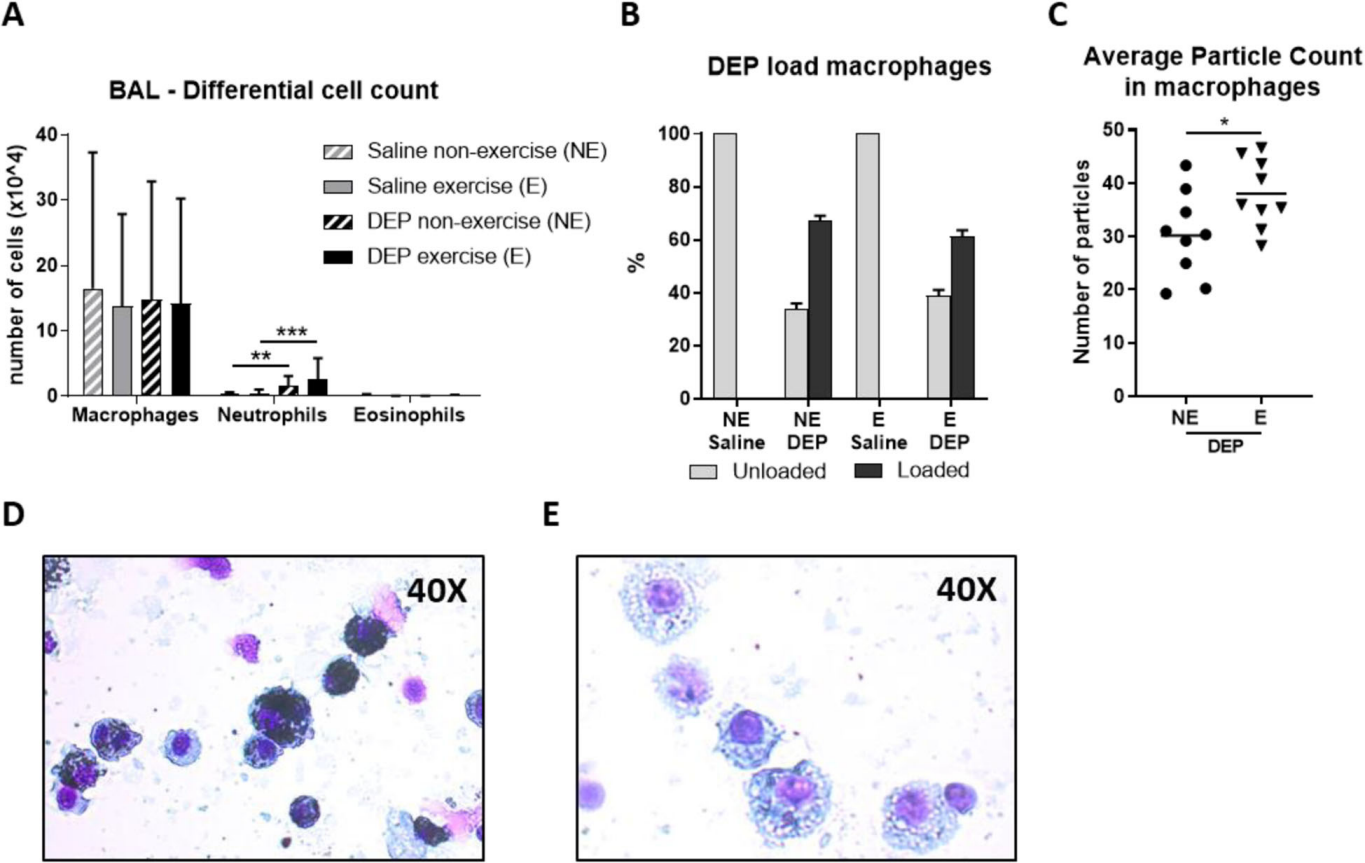
Broncho-alveolar lavage (BAL) and DEP-uptake. **a** Total number of macrophages, neutrophils and eosinophils was counted in bronchoalveolar lavage fluid of experiment 1 and 2. **p < 0.01 and ***p < 0.001 (One-Way ANOVA). **b** The percentage of macrophages loaded with DEP was counted. For each mice of experiment 1 and 2, 100 macrophages were counted in duplicate and an average of the duplicates was calculated per mice. Data is shown as mean with SD in (**a** and **b**) (*n* = 20–21 mice per group). **c** The number of particles taken up by the macrophages was calculated for each mice of experiment 1. Hundred macrophages per mice were counted and an average of the 100 macrophages per mice was calculated, shown in (**c**). *p < 0.05 (Unpaired t-test) (*n* = 8–9 mice per group). **d** Macrophages in bronchoalveolar lavage fluid loaded with DEP, representing DEP/E mice (40 X amplification). **e** Macrophages in bronchoalveolar lavage fluid, representing Sal/E mice. (40 X amplification)

The differential cell count of the bronchoalveolar lavage shows a significant influx of neutrophils in DEP exposed mice, without influence of exercise (Fig. 5a). We also quantified the DEP uptake by the macrophages. Firstly, by counting the number of macrophages loaded with DEP particles, which did not show any difference between DEP/E and DEP/NE mice (Fig. 5b). We also assessed the number of particles taken up by the macrophages, showing that the macrophages of exercising mice (DEP/E) had taken up significantly more DEP particles compared to the non-exercised mice (DEP/NE) (*p* = 0.0364) (Fig. 5c).

### Lung immune response

**Fig. 6:**
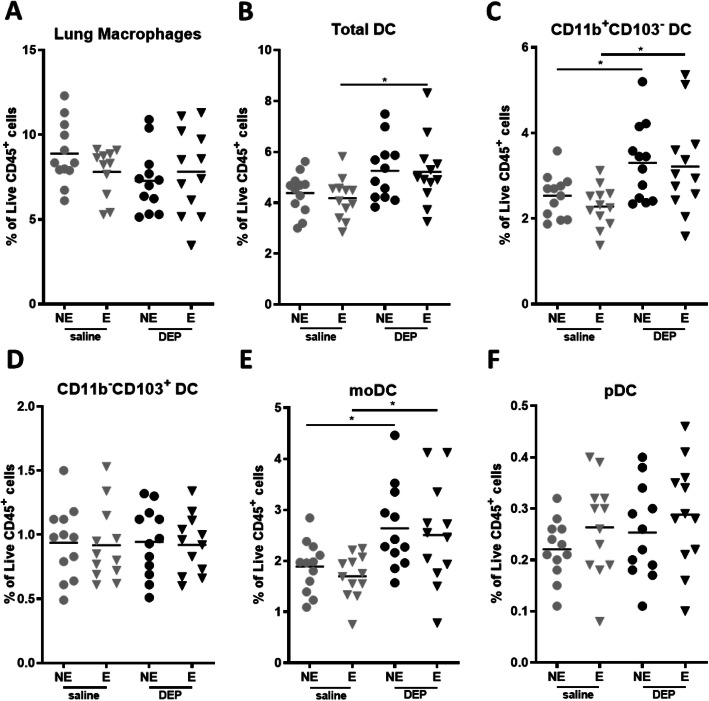
Dendritic cells in lung tissue. Antigen presenting cells in lung tissue were measured using flow cytometry. Cells were analyzed as **a** auto fluorescent macrophages, **b** CD45^+^ low auto fluorescent MHCII^+^CD11c^+^ DC (Total DC), **c** CD45^+^ low auto fluorescent MHCII^+^CD11c^+^CD11b^+^CD103^−^ conventional DC (CD11b^+^ cDC or cDC2), **d** CD45^+^ low auto fluorescent MHCII^+^CD11c^+^CD11b^−^CD103^+^ cDC (CD103^+^ cDC or cDC1), **e** CD45^+^ low auto fluorescent MHCII^+^CD11c^+^CD11b^+^CD64^+^ monocyte derived DC (moDC) and **f** CD45^+^ low auto fluorescent MHCII^+^CD11c^+^SiglecH^+^ plasmacytoid DC (pDC). Data is shown as mean with SD (*n* = 12 mice per group. *p < 0.05 (Two-Way ANOVA). Detailed gating strategy available in supplementary Figure S3

Figure 6 shows the cellular immune response in lung tissue, induced by DEP exposure and/or exercise. The percentage of lung macrophages did not differ between the experimental groups (Fig. 6a). The percentage of the total DC population in the lung was increased in DEP exposed mice compared with saline exposed mice (Two-way ANOVA: *p* = 0.0030) but only reached significance in the exercised mice (*p* < 0.05 for Sal/E compared with DEP/E, Fig. 6b). The DC subpopulations showed a significant increased percentage of lung CD11b^+^CD103^−^ DC and moDC (CD11b^+^CD103^−^CD64^+^) for Sal/NE compared with DEP/NE and for Sal/E compared with DEP/E), respectively (Fig. 6c and e). CD11b^−^CD103^+^ DC and pDC percentages were not significantly changed (Fig. 6d and f). We also evaluated the percentages of the total ILC population and associated subpopulations (ILC1, ILC2, NCR^+^ ILC3 and NCR^−^ ILC3), which were not altered by DEP exposure and/or exercise (Appendix A Fig. S6).

To have a better understanding of the underlying pathways, we measured cytokine levels in BAL fluid. DEP exposure significantly increased the release of GM-CSF, IL-13, KC, TNF-α and MCP-1 into the BAL fluid; while exercise did not influence the cytokine levels (Appendix A Table S3).

### Airway permeability and integrity

**Fig. 7:**
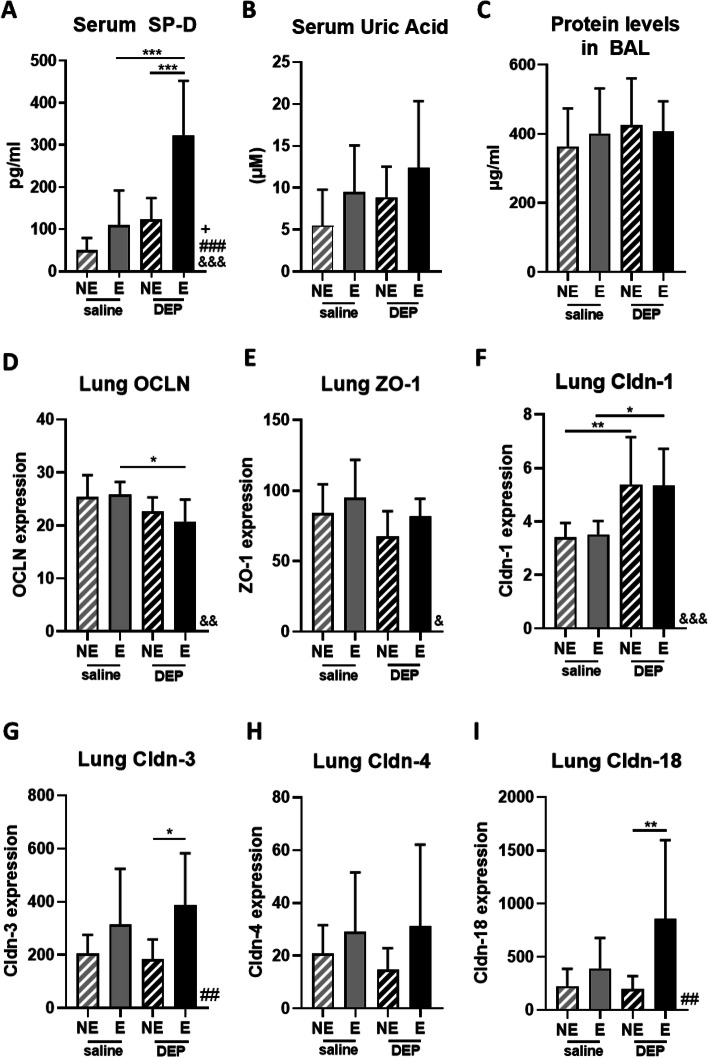
Airway permeability and integrity. **a** Surfactant protein D (SP-D) concentrations in serum were measured using ELISA (R&D Systems). **b** Uric acid concentrations in serum were measured using ELISA (ThermoFisher). **c** Protein levels in bronchoalveolar lavage fluid were measured using a Bradford assay (Biorad). **d** OCLN mRNA expression, **e** ZO-1 mRNA expression, **f** Cldn-1 mRNA expression, **g** Cldn-3 mRNA expression, **h** Cldn-4 mRNA expression and **i** Cldn-18 mRNA expression in lung tissue were measured using RT-qPCR. Data shown as mean with SD (*n* = 9 mice per group). *p < 0.05, **p < 0.01, ***p < 0.001 comparing individual groups. & = DEP-effect, # = Exercise-effect, + = Interaction-effect (Two-Way ANOVA)

Serum SP-D levels were significantly increased in DEP/E mice, compared to the other groups (*p* < 0.001, Fig. 7a). A significant interaction effect between exposure and exercise was shown by Two-Way ANOVA analysis (*p* = 0.0252). Serum UA (Fig. 7b) and total protein levels in BAL fluid (Fig. 7c) were not different between the four groups.

The epithelial integrity was assessed by determining the tight junction expression of OCLN, ZO-1 and Cldn-1, Cldn-3, Cldn-4 and Cldn-18 in lung tissue. OCLN mRNA expression was significantly lower in DEP/E mice compared to DEP/NE mice and Sal/NE mice (*p* < 0.05, Fig. 7d). Cldn-1 mRNA expression was significantly increased in DEP exposed mice compared with saline exposed mice, independently from exercise (*p* < 0.01 for Sal/NE vs. DEP/NE mice and *p* < 0.05 for Sal/E vs DEP/E mice, DEP-effect: *p* < 0.001, Two-way ANOVA Fig. 7f). In DEP exposed mice that performed exercise, Cldn-3 and Cldn-18 mRNA expression was significantly increased compared to non-exercised mice (Fig. 7g and i) (*p* < 0.05 for Cldn-3, *p* < 0.01 for Cldn-18, Exercise-effect of Cldn-3 and Cldn-18: *p* < 0.01, Two-way ANOVA).

### Histology

Lung histological analysis showed that there was no epithelial damage, nor interstitial airway inflammation present; neither in the exercised mice nor in the DEP exposed mice. However, diesel exhaust particles were abundantly present in both the airway lumen and tissue of DEP exposed mice, shown in Appendix A Fig. S7.

### Reversibility of exercise- and DEP-induced effects

In a third experiment, we evaluated if the exercise- and/or DEP-induced effects would sustain over a longer period. Therefore, we added an extra 3 weeks of rest (no exercise and no instillation) to the original protocol and evaluated lung function, airway inflammation and immune responses at day 45. After 3-weeks of rest, the airways were much less sensitive to methacholine, as shown by the overall higher PC20 values in both saline and DEP exposed mice (Appendix A Fig. S8A). Yet at day 45, DEP-induced neutrophilic inflammation and DEP loaded macrophages were still present (Appendix A Fig. S8B and S8C), but the number of DEP loaded macrophages was significantly decreased at day 45 in comparison with day 24 (Appendix A Fig. S8C). Most cytokine levels in BAL fluid were decreased to the concentration of the control mice, except for KC and TNF-α. DEP exposed mice showed decreased KC and TNF-α levels at day 45 compared with day 24, but were still significantly higher compared to saline exposed mice at day 45. Surprisingly, IL-13 levels were reduced in both DEP exposed groups while both saline exposed groups showed a significant higher concentration of IL-13 at day 45 compared with day 24. MCP-1 levels were significantly decreased in all groups at day 45 in comparison with day 24 (data shown in Appendix A Table S4). The concentrations of serum SP-D after 3 weeks without exercise and DEP exposure were similar in all treatment groups (data not shown).

## Discussion

**Fig. 8:**
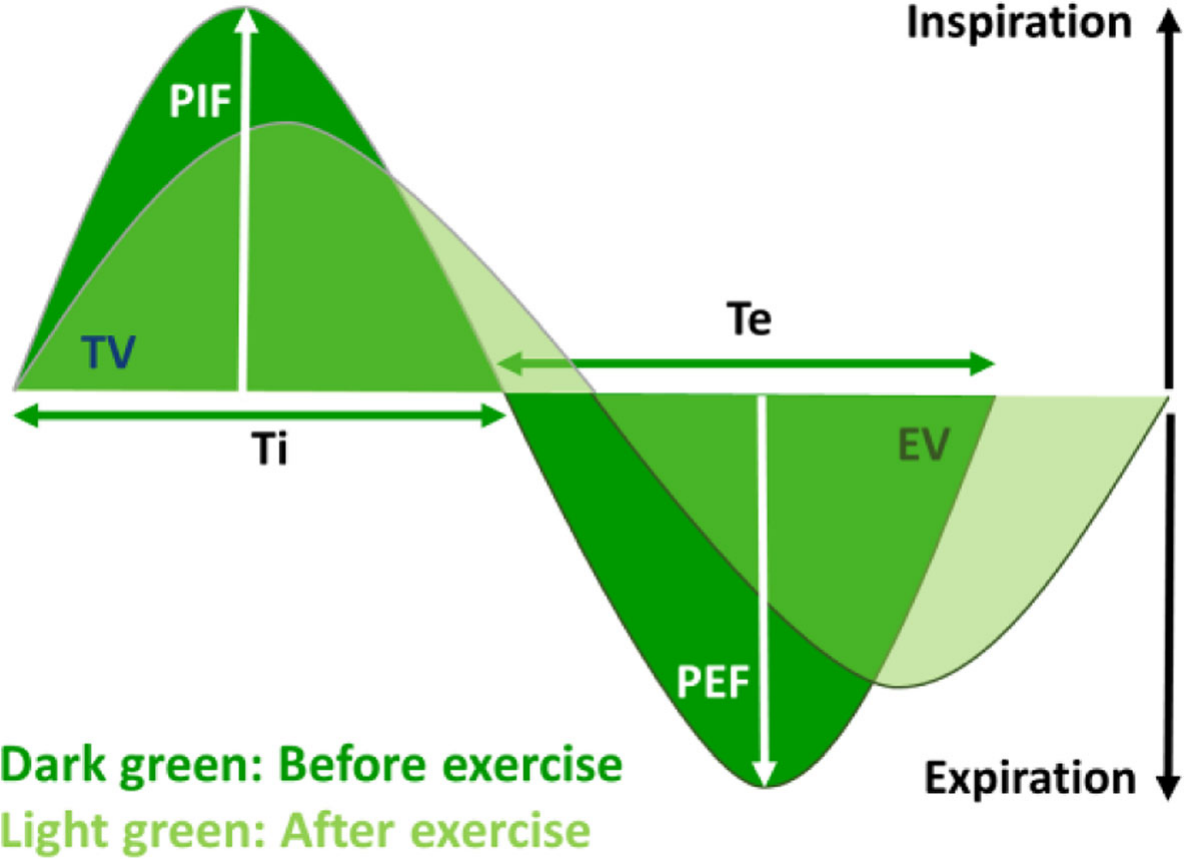
Breathing pattern pre- and post-exposure. Based on the breathing parameters measured pre and post exposure and running, a breathing pattern was reconstructed. Dark green indicates the breathing pattern before exercise and light green after exercise. Ti: inspiratory time, Te: expiratory time, PIF: peak inspiratory flow, PEF: peak expiratory flow, TV: tidal volume, EV: expiratory volume

A recent meta-analysis showed that exposure to air pollution during regular outdoor exercise is associated with an increased risk for cardiopulmonary problems and immune alterations, in both elite athletes and the general population [17]. A better understanding of the impact of outdoor exercise is therefore essential. We studied for the first time the effect of intensive sub-maximal exercise and diesel exhaust particle (DEP) exposure on the lungs of mice. The main findings are shown in Table 1. We showed that intensive exercise leads to an acute change of the breathing pattern of mice, in particular by an increased inspiratory and expiratory time and a decreased peak inspiratory and expiratory flow (Fig. 8). These changes might resemble acute exhaustion. These acute changes were not influenced by the prior DEP exposure, but were getting more pronounced as exercise days progressed, indicating that there is a cumulative effect of repeated exercise. These exercise-induced respiratory changes did not result in airway hyperreactivity (AHR), which was even lower in all exercising mice, suggesting a protective effect of the sub-maximal exercise. We do need to consider that all mice were daily instilled with vehicle or DEP solution, which is probably accumulating in the lungs and is possibly better eliminated in the exercised mice due to the high ventilation. The positive effect of the intensive exercise on AHR is not a real anti-inflammatory effect but rather a physiological adaption of the airways, since the DEP instillations lead to a neutrophilic airway inflammation in both exercised and non-exercised mice. In addition, we did not find changes in pro- and anti-inflammatory cytokine levels in broncho-alveolar lavage fluid, which could explain the reduced airway hyperreactivity after 3 weeks of running. At a molecular level, we showed that submaximal exercise and DEP exposure induce changes at the airway epithelial barrier. OCLN and Cldn-1 expression were altered by DEP exposure, whereas Cldn-3, Cldn-4 and Cldn-18 were significantly increased by the submaximal exercise. These tight junction alterations were accompanied with increased concentrations of club cell produced SP-D in serum, especially in DEP exposed exercised mice. Yet, lung histology revealed no signs of epithelial shear stress or damage. Our findings show that submaximal exercise and low dose DEP exposure induce substantial molecular changes at the airway epithelial barrier, even before physiological effects in the airways are measurable.

**Table 1:** Main findings

	DEP effect	Exercise effect	Interaction effect
**Early ventilatory response**	–	Altered breathing pattern	–
**Airway hyperreactivity**	–	**↓**	–
**Airway inflammation**			
*BAL Neutrophils*	**↑**	–	–
**DC recruitment**			
*CD11b*^*+*^*CD103*^*−*^ *DC*	**↑**	–	–
*moDC*	**↑**	–	–
**Airway permeability**			
*Serum SP-D*	**↑**	**↑**	**↑↑**
*Serum Uric Acid*	–	**↑**	–
*BAL protein levels*	–	–	–
**Airway integrity**			
*OCLN mRNA expression*	**↓**	–	–
*ZO-1 mRNA expression*	**↓**	–	–
*Cldn-1 mRNA expression*	**↑**	–	–
*Cldn-3 mRNA expression*	–	**↑**	–
*Cldn-4 mRNA expression*	–	–	–
*Cldn-18 mRNA expression*	–	**↑**	–

Table 1 shows the major changes induced by DEP exposure (independently from exercise), by exercise (independently from DEP exposure) and by the interaction between DEP exposure and exercise. ↑ represents a significant increase in comparison to the non-exposed or non-exercised groups and ↓ represents a significant decrease in comparison to the non-exposed or non-exercised groups

Only a few studies have investigated the effect of exercise on airway inflammation and immune responses in mice. Vieira et al. showed a significant decrease of the DEP-induced inflammation after 5 weeks of treadmill training, which was not the case in our study. Probably, the exercise intensity plays a role in this issue, as Vieira et al. used an aerobic training protocol (50% of the maximum speed), while we used a more intense submaximal training protocol (70–80% of the maximum speed) [26]. Moreover, we studied cytokine levels in BAL fluid and showed a DEP-induced production of GM-CSF, IL-13, KC, TNF-α and MCP-1. Cytokine levels in BAL were also not reduced after the submaximal training protocol, which is in contrast with KC and TNF-α levels in the aerobic model of Vieira et al. [26].

To understand the involved pathways in exercise and DEP-induced effects, we studied the innate immune system. More specifically focussing on dendritic cells (DC), which are an important bridge between the innate and adaptive immune system, and innate lymphoid cells (ILC), which are activated via the epithelium independently from the adaptive immune system. DC are key players in sensing the environmental triggers and can be divided into several DC subsets. CD103^+^CD11b^−^ DC are located closely underneath the epithelial layers, reaching their dendrites between the epithelial cells, and activate the Th1 response, whereas CD103^−^CD11b^+^ DC are found lower in the subepithelial layers and are thought to be essential for the induction of a Th2 response [27, 28]. The total number of DC in lung tissue was significantly increased in DEP exposed mice in our model, irrespective of the exercise training. More specifically, an increase of CD11b^+^CD103^−^ DC and moDC (CD11b^+^CD103^−^CD64^+^), accompanied with increased levels of MCP-1 (monocyte and dendritic cell attractant) and IL-13 (type 2 cytokine) were detected after DEP exposure. While Th1 inducing CD11b^−^CD103^+^ DC were not influenced by DEP-exposure, explaining the unchanged Th1-related cytokine levels of IFN-γ in BAL fluid. Provoost et al. showed similar results, with an increase of CD11b^+^ DC and MCP-1 levels in BAL fluid. However, they also showed increased levels of CD11b^−^ DC after DEP exposure, which was not the case in our model [27]. This might be due to the 10-fold higher DEP concentration used by Provoost et al. DC can also be activated by epithelial-derived mediators, such as IL-25, IL-33, TSLP, GM-CSF, IL-1β, IL-6 and IL-8 that are released upon binding of allergens, pathogens and pollutants on pattern recognition receptors on the epithelial cells or after epithelial damage [28]. In our model, GM-CSF was significantly increased in the DEP-exposed mice [20, 27]. KC, a chemoattractant and activator of neutrophils, was also significantly increased after DEP-exposure, confirming multiple in vivo and in vitro studies investigating air pollution exposure on the airways. TNF-α, released from activated macrophages and able to induce cytokine release from the epithelium, was significantly higher in mice that were exposed to DEP [20]. Histological analysis revealed clear endocytosis of diesel particles by the macrophages, confirming their activity. IL-33, IL-1β and IL-17A levels were not changed in our model. Innate lymphoid cells (ILC) are part of the innate immune system and their subsets are seen as the counterparts of the adaptive T cell subpopulations, as they produce similar cytokine patterns [29]. Several studies have investigated the effect of particulate matter on ILC subpopulations in the airways, especially on ILC2 [19]. De Grove et al. and Lu et al. showed that DEP-exposure resulted into increased numbers of ILC2, along with Th2 cells, in BAL fluid of mice exposed to DEP and house dust mite or ovalbumin, respectively [30–32]. Until now, it is not clearly known to which extent ILC1 and ILC3 can be modulated by PM exposure [20, 33]. We found no changes in the ILC subsets induced neither by DEP nor by exercise. In the above described models, changes in the ILC subsets were only seen in allergic HDM and OVA models in which DEP acts as an adjuvant for the type 2-activating allergens [19].

Epithelial barrier function and integrity are essential in the protection against foreign noxious particles. We have evaluated proteins, which pass the epithelial barrier in case of barrier disruption, and tight junctions on epithelial cells, which are essential for barrier maintenance at a molecular level. Serum surfactant protein D (SP-D), as a marker for the leakage from the airways into the circulation [34], was significantly increased in DEP exposed mice that followed the intensive exercise protocol, suggesting that the combination of intensive exercise and DEP exposure results in epithelial damage. Yet, serum uric acid, which is released by damaged epithelial cells, and protein levels in BAL, a marker for leakage from the circulation into the airways, were not altered. This indicates that the increased SP-D levels are not caused by epithelial damage, but probably are caused by SP-D overproduction of the club cells. SP-D overproduction is possibly a compensatory reaction of the airways to protect them from acute injury and to control the macrophage activation [35, 36]. On the level of epithelial barrier integrity, we found that OCLN expression in lung tissue was significantly decreased after DEP exposure, confirming the reduced presence of occludin in human airway epithelial cells after in vitro PM_10_ and DEP exposure [37]. Cldn-1 expression was significantly increased after DEP exposure, which is in contrast with the Cldn-1 degradation described in literature [38]. Intensive exercise mainly induced an upregulation of Cldn-3, − 4 and − 18, which was more pronounced in DEP exposed exercised mice in comparison with saline exposed exercised mice. Mitchell et al. showed that increased Cldn-3 expression is associated with decreased permeability [39], which we could not confirm. Cldn-4 expression was upregulated as a protective effect after acute injury in the ventilator-induced lung injury murine model of Wray et al., in which they applied air at high ventilation rates into the lungs [40]. These findings support the exercise-induced Cldn-4 alterations in our model, as the intensive exercise also requires high ventilation rates. Cldn-18, one of the major tight junctions of the alveolar epithelium, which is suggested to be regulated by IL-13 [41], was increased after intensive exercise. However, this was not accompanied with changes in IL-13, since IL-13 was mainly influenced by DEP exposure and not by exercise in our model. Our data show that exercise and DEP-exposure resulted in a significant dysregulation of the tight junction composition of the airway epithelium, which is known to be associated with lung diseases.

For our experiments, we have selected a relatively low concentration of DEP (0.2 mg/ml or 10 μg). This concentration is at least 10-fold lower than DEP concentrations used in other studies, which are ranging between 1 to 10 mg/ml [26, 31, 42–44]. DEP was administrated via intranasal instillation (50 μL), for which it is described that only a 50% from the volume reaches the lower airways and alveolar regions, resulting in an estimated dose of only 5 μg reaching the lower airways [45]. To prove the relevance of the DEP exposure concentration in our model, we calculated the dose of DEP inhaled by humans during a 30 min light exercise session (minute volume of 18 L/min) in an area with an environmental PM_2.5_ concentration of 14.5 μg/m^3^, which is based on a 14-day average of the PM_2.5_ concentration in Leuven (Belgium) (“Intergewestelijke Cel voor het Leefmilieu (IRCEL)”) [46]. This results in an estimated dose of 7.5 μg PM_2.5_, which is in the range of the concentration used in our murine model. Moreover, this concentration is also in line with the limit values imposed by the WHO (25 μg/m^3^) [47]. Our calculations show that our findings are relevant for daily exposure to air pollution in Flanders, which is an important benefit of our study. Nevertheless, exercise on itself induced only limited changes in the airways. This raises the question if protocol intensity and duration were high and long enough, which features an important limitation of the model.

## Conclusion

In conclusion, we evaluated the effect of submaximal exercise and diesel exhaust particle exposure on the airways of non-allergic mice. DEP exposure resulted in a typical neutrophilic airway inflammation associated with DC recruitment and elevated pro-inflammatory cytokine levels in BAL fluid. Submaximal exercise induced a significant acute exhaustion, which progressed over time. Furthermore, our submaximal intense exercise model could not confirm the anti-inflammatory effects reducing the air pollution induced pulmonary inflammation, as seen with aerobic exercise in previous studies. Both DEP exposure and intensive exercise induced significant alterations in the expression of OCLN, Cldn-1, − 3, − 4 and − 18, resulting in an important tight junction dysregulation. These findings provide evidence that outdoor sports activities, at high intensity in combinations with high outdoor pollution, can induce changes at a molecular level, which may lead to respiratory problems.

## Supplementary Information

**Additional file 1.**

**Additional file 2.**

## Data Availability

Data sharing is not applicable to this article as no datasets were generated or analysed during the current study.
