# Supplementary material for: Differential effects of intense exercise and pollution on the airways in a murine model
Source: Part Fibre Toxicol. 2021 Mar 15;18:12. doi: 10.1186/s12989-021-00401-6 (PMC7962283; doi:10.1186/s12989-021-00401-6)
Supplement: Supplementary file 1 — Additional file 1. [file 12989_2021_401_MOESM1_ESM.docx]

**Supplementary tables and figures**

Figure S1:

**
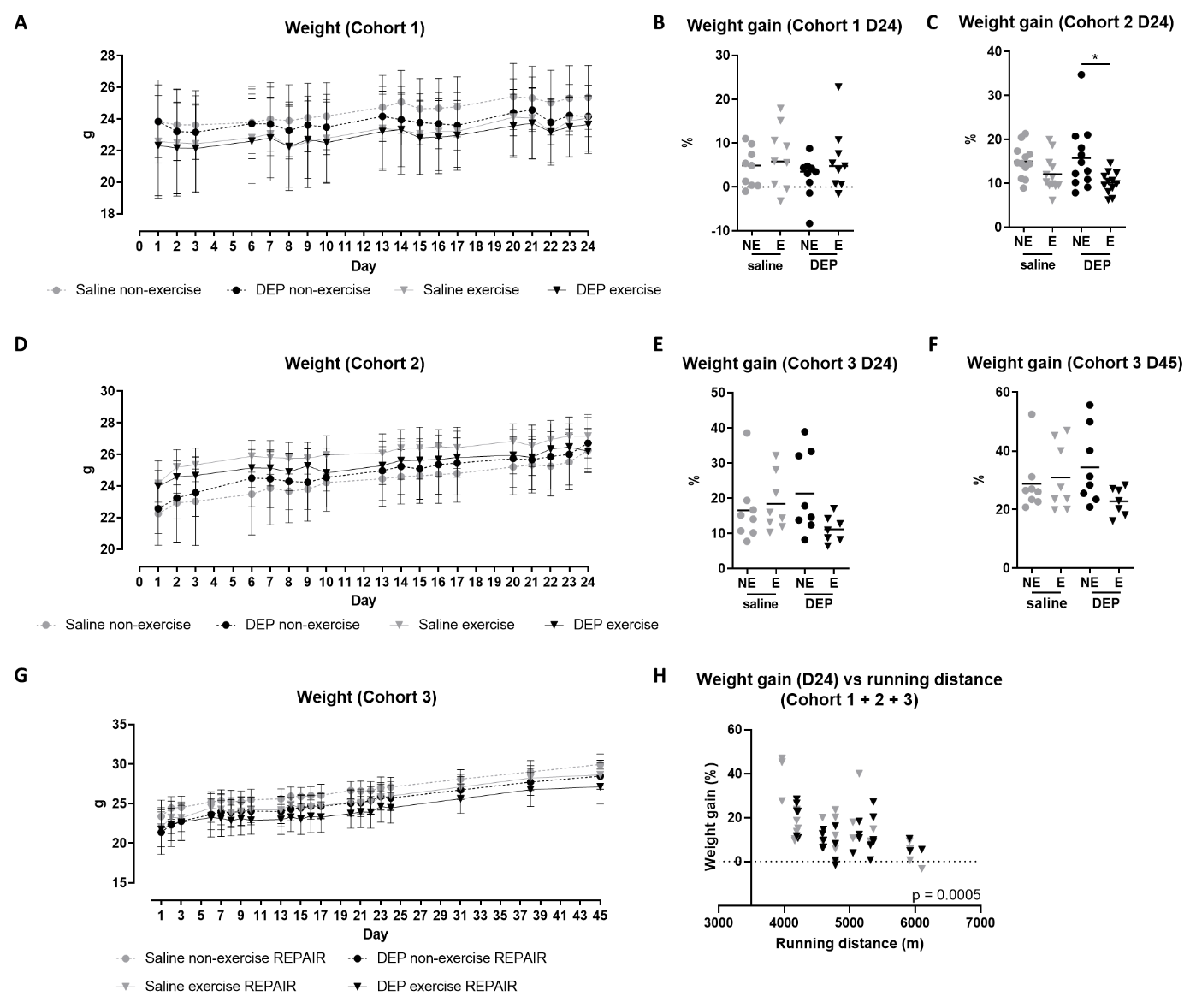
**

**Figure S1: Body weight and weight gain. (A, D and G)** Body weight during the protocol of cohort 1, 2 and 3, respectively. **(B, C and E)** Body weight gain (%) at day 24 of cohort 1, 2 and 3, respectively. (Kruskal-Wallis test, *p < 0.5). **(F)** Body weight gain (%) at day 45 of cohort 3. **(H)** Correlation between body weight gain (%) and total running distance. (Spearman correlation)

Table S1:

| **Day 1 and 2** Acclimatisation | - 5 min. 0 m/min  - 10 min. 6 m/min |
| --- | --- |
| **Day 3**  Endurance Test | - 5 min. 3 m/min  - Increase of 1 m/min every minute until exhaustion |
| **Day 6 – 10**  Running session at 70% | - 5 min. 6 m/min  - Increase of 1 m/min every minute until 70% of the maximum speed  - Continue at 70% of maximum speed until a total of 30 minutes running is completed |
| **Day 13 – 17**  Running session at 75% | - 5 min. 6 m/min  - Increase of 1 m/min every minute until 75% of the maximum speed  - Continue at 75% of maximum speed until a total of 30 minutes running is completed |
| **Day 20 – 24**  Running session at 80% | - 5 min. 6 m/min  - Increase of 1 m/min every minute until 80% of the maximum speed  - Continue at 80% of maximum speed until a total of 30 minutes running is completed |

**Table S1: Detailed submaximal running protocol**

Table S2:

| **Maximal running capacity** | **Completed running distance at the end of the training protocol** | **Number of mice** |
| --- | --- | --- |
| **Experiment 1** | | |
| **15 m/min** | **4210.0 m** | **3** |
| **18 m/min** | **4782.2 m** | **4** |
| **19.5 m/min** | **5049.4 m** | **3** |
| **21 m/min** | **5316.5 m** | **3** |
| **25 m/min** | **5920.0 m** | **3** |
| **27 m/min** | **6101.7 m** | **2** |
| **Experiment 2** | | |
| **15 m/min** | **4189.0 m** | **5** |
| **16 m/min** | **4160.3 m** | **2** |
| **17 m/min** | **4585.7 m** | **4** |
| **18 m/min** | **4782.2 m** | **3** |
| **19 m/min** | **4971.3 m** | **4** |
| **20 m/min** | **5145.0 m** | **2** |
| **22 m/min** | **5361.1 m** | **2** |
| **25 m/min** | **5913.5 m** | **2** |
| **Experiment 3** | | |
| **14 m/min** | **3966.5 m** | **4** |
| **15 m/min** | **4189.0 m** | **4** |
| **18 m/min** | **4782.2 m** | **3** |
| **20 m/min** | **5145.0 m** | **2** |
| **22 m/min** | **5361.1 m** | **3** |

**Table S2: Maximal running speed and calculated total running distances.**

Each mice performed an endurance test at day 3 to determine their maximal running capacity. A total running distance was calculated for each mice based on the running speed used during the training protocol in week 1, 2 and 3 (respectively 70 %, 75 % and 80 % of the maximal running speed).

Figure S2:


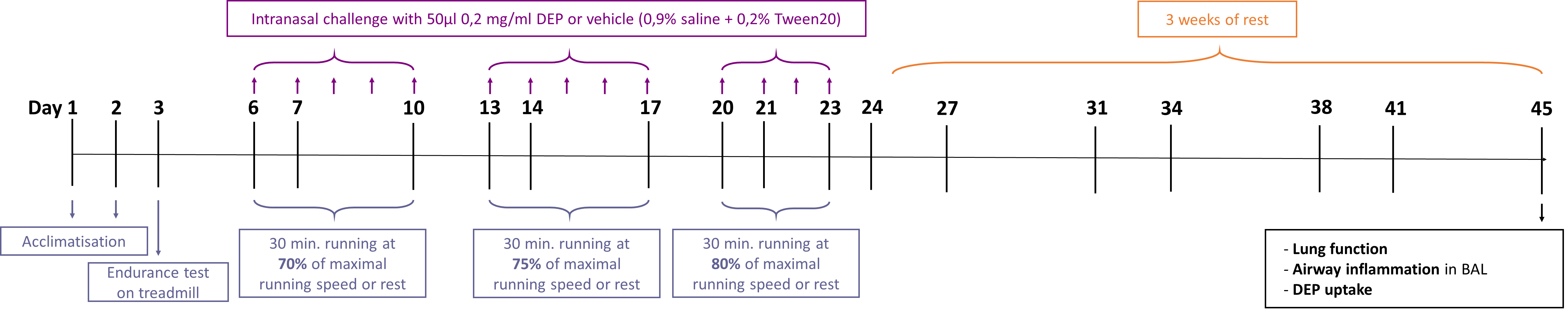


**Figure S2: Study design of experiment 3.**

Figure S3:


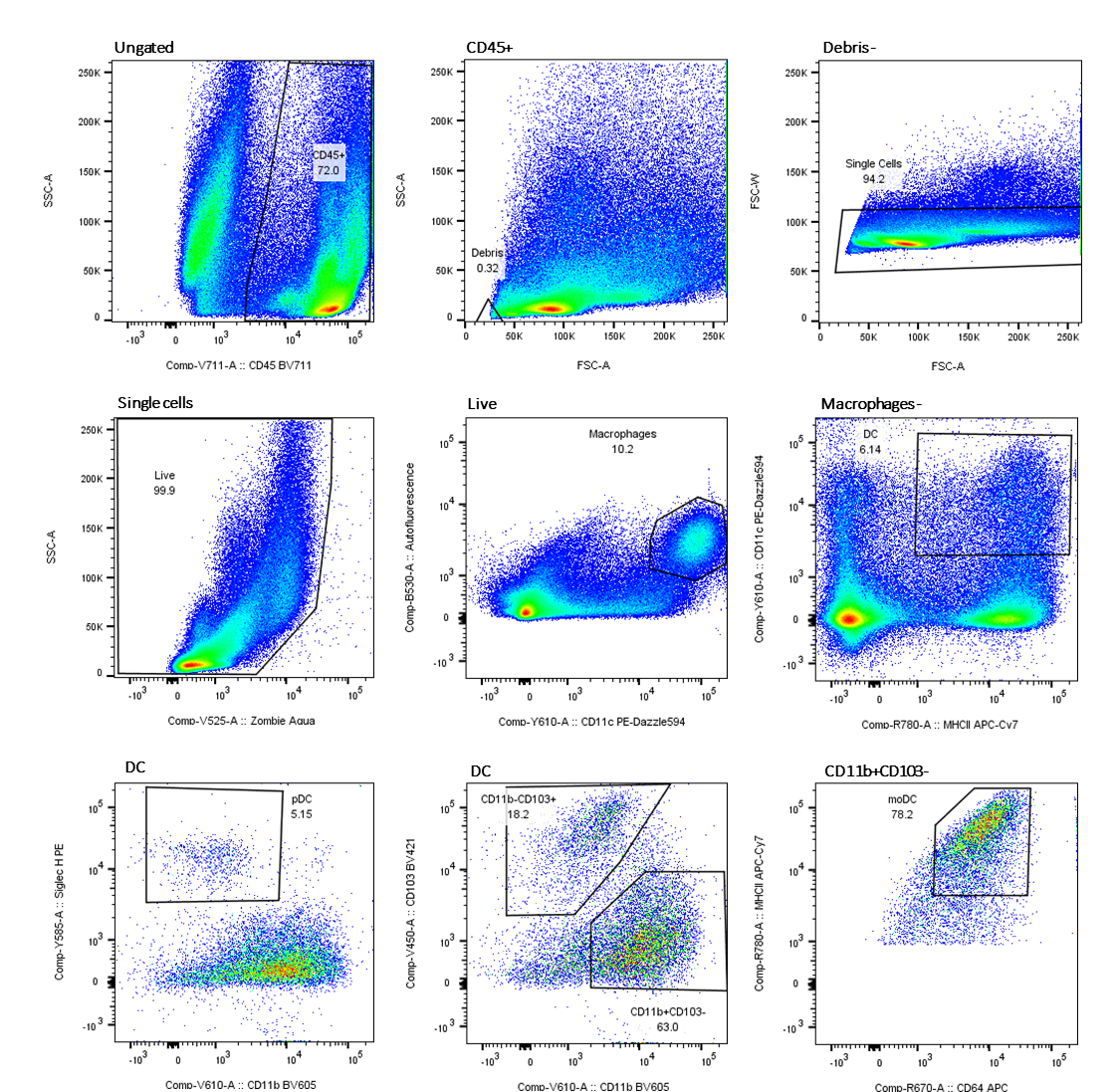


**Figure S3: Representative flow cytometry data and gating strategy for dendritic cell (DC) populations in lung.** Dendritic cells were marked as low-autofluorescent, CD11c^+^ and MHCII^+^. CD11c^+^ and high-autofluorescent cells were marked as macrophages. Plasmacytoid DC (pDC) were gated as CD11^-^ and SiglecH^+^. Conventional dendritic cells (cDC) were subdivided into CD11b^+^CD103^-^ cells and CD11b^-^CD103^+^ cells. Monocyte-derived DC (moDC) were identified as CD11b^+^CD103^-^CD64^+^. Gating was created based on Fluorescence Minus One Controls. FCS, forward scatter; SSC, side scatter. Data shown of DEP/E mice.

Figure S4:


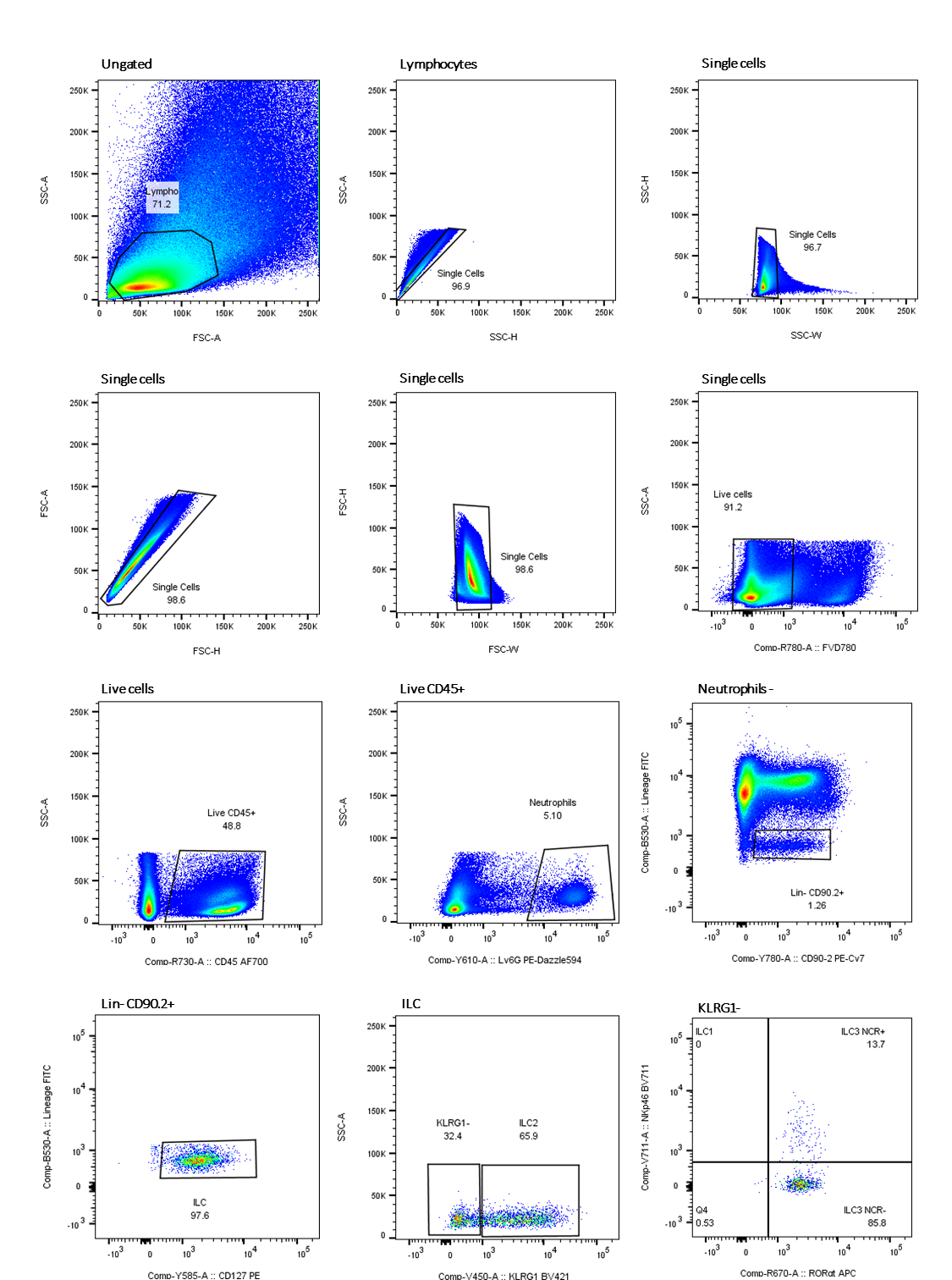


**Figure S4: Representative flow cytometry data and gating strategy for ILC populations in the lung.** Neutrophils were gated as live CD45^+^ and Ly6G^+^ cells. ILC were marked as live CD45^+^, Ly6G^-^, Lin^-^ (CD11b, CD19, CD3e, CD45RB, CD49b, CD5, CD94, TCRγδ and Ter-119), CD90.2^+^ and CD127^+^ cells. ILC2 were marked as KLRG-1^+^, ILC1 were marked as KLRG-1^-^RORγT^-^NKp46^+^, NCR^-^ ILC3 were marked as KRLG-1^-^RORγT^+^NKp46^-^ and NCR^+^ ILC3 as KRLG-1^-^RORγT^+^NKp46^+^. The gating was created based on Fluorescence Minus One Controls. FCS; forward scatter, SSC; side scatter. Data shown of DEP/E mice.

Figure S5:

**A**

**B**

**C**

**D**

**Figure S5: Longitudinal changes of the baseline breathing pattern at day 1, 8, 15 and 22.**

Breathing parameters were measured on day 1, 8, 15 and 22 prior to the exposure and running or rest session using a double chamber plethysmograph (EMKA). **(A)** The average expiratory time (Te) pre-exposure. **(B)** The average peak expiratory flow (PEF) pre-exposure. **(C)** The average expiratory volume (EV) pre-exposure. **(D)** The average tidal volume (TV) pre-exposure. *p < 0.05 (Two-Way ANOVA). Data is shown as mean (n = 7-9 mice per group).

Figure S6:


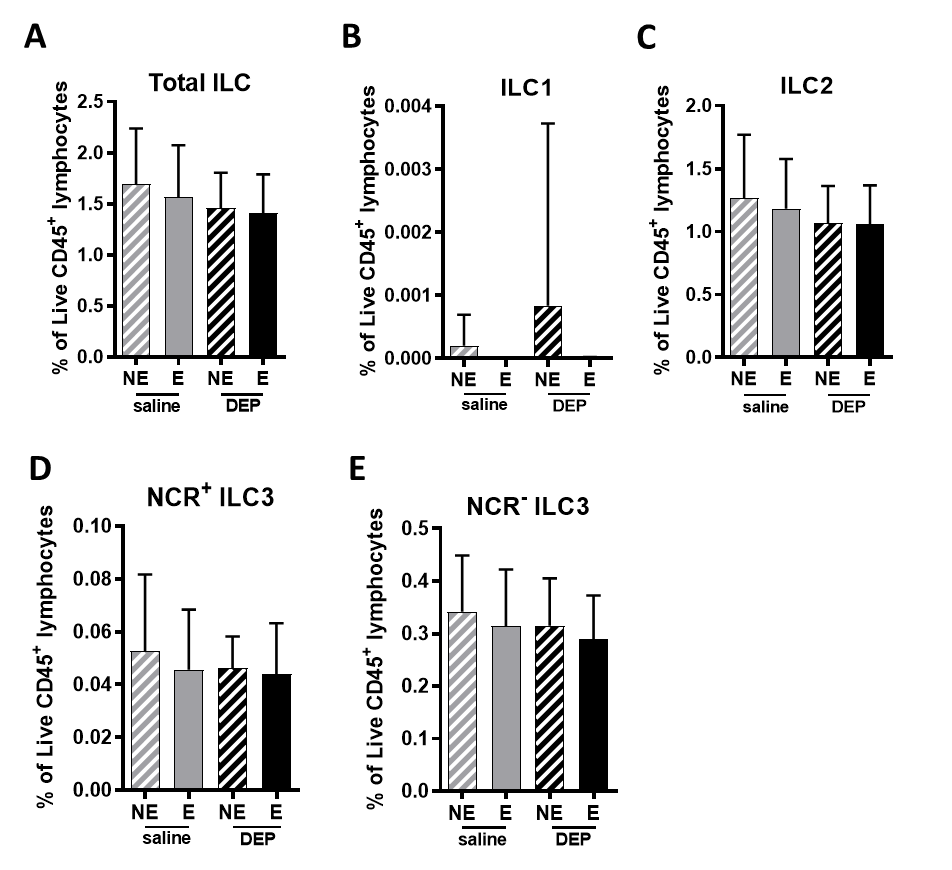


**Figure S6: Innate lymphoid cell (ILC) subpopulations in lung tissue.**

Innate lympoid cells were measured using flow cytometry. Cells were analysed as **(A)** CD45^+^, Lin^-^, CD90.2^+^, CD127^+^ (Total ILC), and subpopulations were characterized as **(B)** KLRG1^-^, NKp46^+^ ILC1, **(C)** KLRG1^+^ ILC2, **(D)** KLRG1^-^, RORγT^+^, NKp46^+^ NCR^+^ILC3 and **(E)** KLRG1^-^, RORγT^+^, NKp46^-^ NCR^-^ILC3. Data is shown as mean with SD (n = 12 mice per group). Detailed gaiting strategy available in supplementary figure S3.

Table S3:

|  | **Sal/NE** | **DEP/NE** | **Sal/E** | **DEP/E** | **Two-Way ANOVA** |
| --- | --- | --- | --- | --- | --- |
| **GM-CSF** | 0.07  (0.03 – 0.12) | **0.43*****  **(0.19 – 0.52)** | 0.14  (0.07 – 0.20) | **0.39****  **(0.18 – 0.48)** | ***  DEP-effect |
| **IFN-γ** | 0.00  (0.00 – 0.01) | 0.00  (0.00 – 0.01) | 0.00  (0.00 – 0.07) | 0.00  (0.00 – 0.06) |  |
| **IL-13** | 0.00  (0.00 – 1.93) | **5.16***  **(0.87 – 9.11)** | 0.47  (0.00 – 5.63) | **6.50**  **(0.00 – 8.91)** | ***  DEP effect |
| **IL-17A** | 0.01  (0.00 – 0.03) | 0.05  (0.00 – 0.26) | 0.01  (0.00 – 0.09) | 0.03  (0.00 – 0.09) |  |
| **IL-1β** | 0.51  (0.00 – 1.19) | 0.82  (0.25 – 1.98) | 0.79  (0.00 – 1.51) | 0.91  (0.70 – 2.67) |  |
| **KC** | 30.49  (27.17 – 43.70) | **64.13*****  **(48.62 – 104.7)** | 28.97  (22.51 – 40.45) | **55.84***  **(34.56 – 74.43)** | ***  DEP effect |
| **TNF-α** | 3.07  (1.69 – 3.31) | **5.30***  **(2.36 – 11.61)** | 2.91  (2.04 – 3.66) | 4.60  (2.33 – 11.71) | ***  DEP effect |
| **IL-17F** | 0.00  (0.00 – 1.62) | 0.27  (0.00 – 1.34) | 0.08  (0.00 – 0.95) | 0.00  (0.00 – 0.14) |  |
| **IL-33** | 2.14  (0.81 – 3.34) | 3.67  (1.37 – 4.92) | 2.02  (1.38 – 5.18) | 1.65  (1.25 – 3.29) |  |
| **MCP-1** | 0.57  (0.00 – 1.74) | **2.78***  **(0.76 – 15.53)** | 0.65  (0.00 – 2.45) | **4.57***  **(0.52 – 16.45)** | ***  DEP effect |
| **MIP-2** | 17.36  (9.27 – 20.58) | 17.32  (11.18 – 26.48) | 11.90  (10.20 – 17.10) | 15.33  (9.88 – 22.09) |  |

**Table S3: Cytokine measurements in BAL fluid.**

Cytokine levels in bronchoalveolar lavage fluid of experiment 1 and 2 were measured, using a U-plex assay (Meso Scale Diagnostics). ***p < 0.001, Two-Way ANOVA. Data is shown as median with IQR (pg/ml) (n = 21/group). (GM-CSF: granulocyte-macrophage colony-stimulating factor, IFN: interferon, IL: interleukin, KC: keratinocyte-derived chemokine, TNF: tumor necrosis factor, MCP: monocyte chemoattractant protein-1, MIP: macrophage inflammatory protein)

Figure S7:


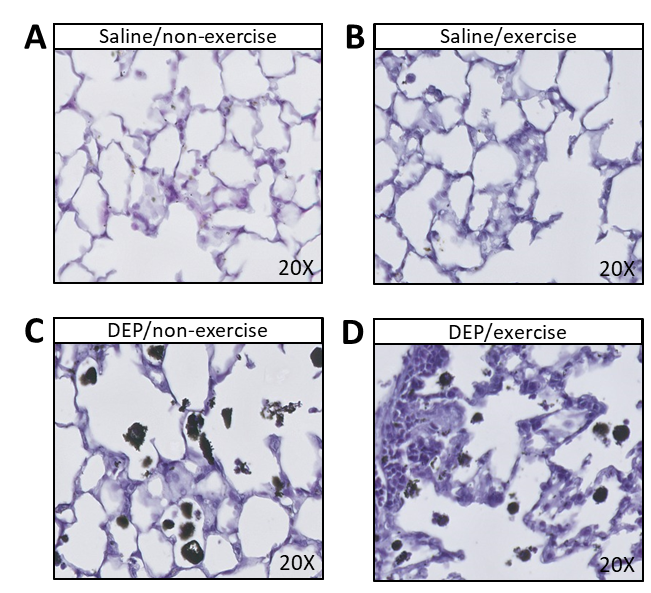


**Figure S7: Histology of lung tissue.**

H&E staining was performed on lung slices of mice sacrificed 24h after the last exercise or non-exercise session. **(A)** Representative image of the normally preserved lung tissue of Sal/NE mice (20X amplification). **(B)** Representative image of the normally preserved lung tissue of Sal/E mice (20X amplification). **(C)** and **(D)** Images of the presence of diesel exhaust particles in respectively DEP/NE mice and DEP/E mice (20X amplification). Diesel exhaust particle are characterised by prominent black pigmented deposits. Sal: saline, DEP: diesel exhaust particle, E: exercise, NE: non-exercise.

Figure S8:


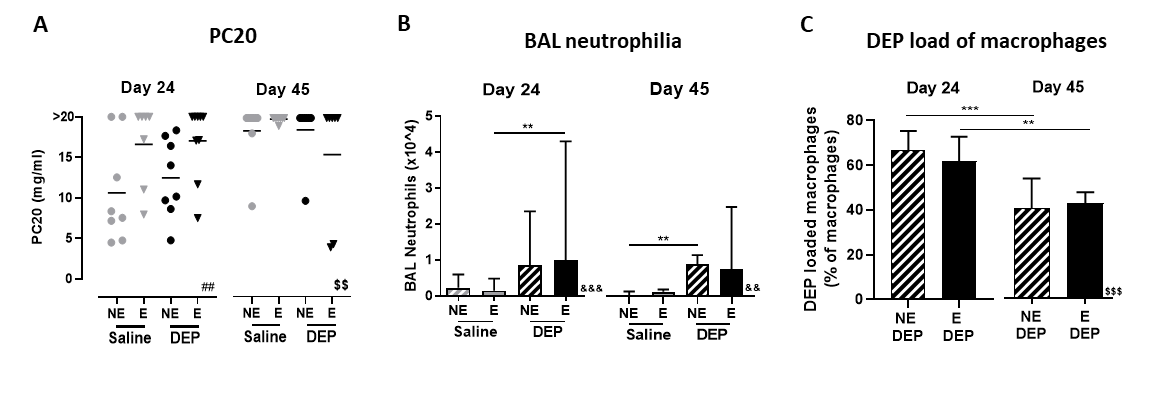


**Figure S8: Reversibility of the exercise and DEP-induced effects.**

Lung function, airway inflammation and DEP-uptake were measured, in which a 3 weeks rest period (no exercise and no instillations) was added to the standard protocol to evaluate the reversibility of the exercise and DEP-induced effects. **(A)** Airway hyperreactivity, shown as PC20, at day 24 (standard protocol, as shown in Fig. 4F) and day 45 (reversibility protocol). Exercise-effect; ##: p < 0.01 (Two-Way ANOVA). Reversibility effect; $$: p < 0.01 (Three-Way ANOVA). **(B)** Airway inflammation, shown as the total amount of neutrophils in BAL, at day 24 (standard protocol, Fig. 5A) and day 45 (reversibility protocol). **p < 0.01, DEP-effect; &&&: p < 0.001, &&: p < 0.01 (Two-Way ANOVA). **(C)** The number of DEP loaded macrophages was counted in the DEP-exposed mice at day 24 (standard protocol, Fig. 5B) and day 45 (reversibility protocol). **p < 0.01, ***p < 0.001, Reversibility effect; $$$: p < 0.001 (Two-Way ANOVA) (n = 7-8 mice /group). Data is shown as individual data points with mean for PC20, as median with IQR for BAL neutrophilia and as mean with SD for DEP loaded. DEP: diesel exhaust particles, PC20: provocative concentration that induces a 20% in FEV_0.1_(%), BAL: broncho-alveolar lavage, NE: non-exercise, E: exercise, IQR: interquartile range, SD: standard deviation.

|  | **Sal/NE  (Day 24)** | **Sal/NE + REPAIR (Day 45)** | **DEP/NE  (Day 24)** | **DEP/NE + REPAIR (Day 45)** | **Sal/E (Day 24)** | **Sal/E +  REPAIR (Day 45)** | **DEP/E (Day 24)** | **DEP/E + REPAIR (Day 45)** |
| --- | --- | --- | --- | --- | --- | --- | --- | --- |
| **GM-CSF** | 0.07  (0.03 – 0.12) | 0.16  (0.02 – 0.34) | 0.43  (0.19 – 0.52) | 0.24  (0.17 – 0.27) | 0.14  (0.07 – 0.20) | 0.27  (0.22 – 0.40) | 0.39  (0.18 – 0.48) | 0.19  (0.08 – 0.40) |
| **IFN-γ** | 0.00  (0.00 – 0.01) | Undetectable | 0.00  (0.00 – 0.01) | 0.04  (0.00 – 0.14) | 0.00  (0.00 – 0.07) | 0.00  (0.00 – 0.14) | 0.00  (0.00 – 0.06) | 0.00  (0.00 – 0.06) |
| **IL-13** | **0.00**  **(0.00 – 1.93)** | **8.61***  **(2.42 – 14.35)** | 5.16  (0.87 – 9.11) | 0.00  (0.00 – 5.37) | **0.47**  **(0.00 – 5.63)** | **9.83*****  **(4.74 – 22.50)** | 6.50  (0.00 – 8.91) | 1.45  (0.00 – 18.20) |
| **IL-17A** | 0.01  (0.00 – 0.03) | 0.00  (0.00 – 0.01) | 0.05  (0.00 – 0.26) | 0.04  (0.00 – 0.06) | 0.01  (0.00 – 0.09) | 0.02  (0.00 – 0.05) | 0.03  (0.00 – 0.09) | 0.01  (0.00 – 0.04) |
| **IL-1β** | 0.51  (0.00 – 1.19) | 0.32  (0.27 – 0.55) | 0.82  (0.25 – 1.98) | 0.89  (0.65 – 1.07) | 0.79  (0.00 – 1.51) | 0.17  (0.00 – 0.42) | 0.91  (0.70 – 2.67) | 0.45  (0.27 – 0.83) |
| **KC** | 30.49  (27.17 – 43.70) | 12.18  (7.81 – 12.94) | **64.13**  **(48.62 – 104.7)** | **29.33*****  **(19.10 – 33.37)** | 28.97  (22.51 – 40.45) | 22.32  (15.40 – 38.11) | **55.84**  **(34.56 – 74.43)** | **24.57**  **(19.66 – 34.36)** |
| **TNF-α** | 3.07  (1.69 – 3.31) | 0.59  (0.35 – 0.71) | **5.30**  **(2.36 – 11.61)** | **2.05****  **(0.92 – 2.76)** | 2.91  (2.04 – 3.66) | 0.94  (0.47 – 1.24) | **4.60**  **(2.33 – 11.71)** | **1.05**  **(0.77 – 1.31)** |
| **IL-17F** | 0.00  (0.00 – 1.62) | Undetectable | 0.27  (0.00 – 1.34) | Undetectable | 0.08  (0.00 – 0.95) | Undetectable | 0.00  (0.00 – 0.14) | Undetectable |
| **IL-33** | 2.14  (0.81 – 3.34) | 1.98  (0.92 – 5.05) | 3.67  (1.37 – 4.92) | 2.81  (1.68 – 5.64) | 2.02  (1.38 – 5.18) | 2.50  (1.64 – 4.23) | 1.65  (1.25 – 3.29) | 2.28  (0.58 – 7.92) |
| **MCP-1** | 0.57  (0.00 – 1.74) | 2.55  (0.00 – 6.92) | 2.78  (0.76 – 15.53) | 1.28  (0.00 – 7.51) | 0.65  (0.00 – 2.45) | 1.08  (0.00 – 4.93) | 4.57  (0.52 – 16.45) | 1.00  (0.00 – 6.30) |
| **MIP-2** | **17.36**  **(9.27 – 20.58)** | **3.81***  **(2.71 – 4.57)** | **17.32**  **(11.18 – 26.48)** | **7.53***  **(5.03 – 11.97)** | **11.90**  **(10.20 – 17.10)** | **5.43***  **(4.14 – 6.48)** | **15.33**  **(9.88 – 22.09)** | **6.11**  **(4.94 – 6.97)** |

**Table S4: Cytokine levels in BAL fluid at day 24 and day 45.**

Cytokine levels in bronchoalveolar lavage fluid of experiment 1 and 2 (at day 24) and experiment 3 (at day 45, after an additional 3 weeks rest period) were measured, using a U-plex assay (Meso Scale Diagnostics). A Two-Way ANOVA, with post-hoc Bonferroni’s multiple comparison test was performed to compare the ‘STANDARD’ groups with the ‘REPAIR’ groups. Bold: non-significant trend, *p < 0.05, **p < 0.01, ***p < 0.001, Two-Way ANOVA. Data is shown as median with IQR (pg/ml) (n = 21/group for ‘STANDARD’ groups and n = 8/group for ‘REPAIR’ groups). (GM-CSF: granulocyte-macrophage colony-stimulating factor, IFN: interferon, IL: interleukin, KC: keratinocyte-derived chemokine, TNF: tumor necrosis factor, MCP: monocyte chemoattractant protein-1, MIP: macrophage inflammatory protein)
