# Supplementary material for: Differential effects of intense exercise and pollution on the airways in a murine model
Source: Part Fibre Toxicol. 2021 Mar 15;18:12. doi: 10.1186/s12989-021-00401-6 (PMC7962283; doi:10.1186/s12989-021-00401-6)
Supplement: Supplementary file 2 — Additional file 2. [file 12989_2021_401_MOESM2_ESM.docx]

**Supplementary materials and methods**

Early ventilatory response

The early ventilatory response was measured on days 1, 8, 15 and 22 using a double chamber plethysmography (DCP, Emka Technologies, Paris, France). Mice were restrained and placed in the plethysmograph, composed of a nasal and thoracic chamber, to measure following respiratory parameters; peak inspiratory flow (PIF), peak expiratory flow (PEF), minute volume (MV), tidal volume (TV), breathing frequency (f), inspiratory time (Ti), expiratory time (Te), end inspiratory pause (EIP), end expiratory pause (EEP), resistance (sRaw) and conductance (sGaw). Respiratory parameters were determined based on pressure changes in the nasal and thoracic chamber of the DCP. Before the exercise or non-exercise period, mice were placed in the DCP for acclimatisation (10 min) followed by 5 minutes respiratory measurement. Immediately after the exercise or non-exercise session, mice were placed back in the DCP for another 5 minutes acclimatisation and 5 minutes respiratory measurement. During the measurement period, every 30 seconds data was collected and the average of the 10 datapoints was used for statistical analysis.

Airway and tissue hyperreactivity

Twenty-four hours after the last running session, airway hyperreactivity and lung function parameters were assessed using the FlexiVent™ system (EMKA Technologies - SCIREQ, Montreal, Canada). The system was designed to concurrently measure forced oscillations and forced expiration parameters. Briefly, the system operated with flexiWare™ 7.3 software and was equipped with a FX1 module, a negative pressure forced expiration (NPFE) extension for mice and a small particle size Aeroneb® Lab nebulizer (2.5-4 μm; Aerogen, Galway, Ireland). Mice were anesthetized with an intraperitoneal injection of pentobarbital (120 mg/kg BW, Dolethal ®). Once sufficiently anesthetized, a tracheotomy was performed to insert an 18-gauge metal cannula. Mice were quasi-sinusoidally ventilated with a tidal volume of 10 mL/kg and a frequency of 150 breaths/min to mimic spontaneous breathing.

At the start of the experiment, two successive deep inflations were applied to maximally inflate the lungs to a pressure of 30 cmH2O in order to open the lungs.

Next, to determine airway responsiveness, a protocol to evaluate the airway hyperreactivity was initiated. An aerosol containing increasing concentrations of the bronchoconstricting agent methacholine (MCh) (0, 1.25, 2.5, 5, 10, 20 mg/mL in saline) was nebulized for 10 sec. After the aerosol exposure, the forced oscillation perturbation ‘Quick Prime-3’ (QP3), generating forced oscillations with a frequency between 1-20.5 Hz during 3 sec, was executed five times. The reported central airway resistance (Rn) for each concentration is the average of these five measurements. After the sequence, a NPFE perturbation was performed to measure the forced vital capacity (FVC) and forced expiratory volume in 0.1 seconds (FEV0.1) at each concentration.

If the coefficient of determination (COD) of a measurement was lower than 0.9, the measurement was excluded. For each mouse, Rn and FEV0.1 were plotted against the Mch concentration.

Rn, FVC and FEV0.1 were identically measured prior to any Mch aerosol exposure to determine the baseline values for each mouse. FEV_0.1_ and FVC were used to calculated the Tiffeaneau-index_0.1_ (FEV_0.1_/FVC).

RT-qPCR

Primer and probe sequences for the specific genes were developed in the laboratory of Clinical Immunology using Primer Express (Applied Biosystems, Thermo-Fisher Scientific). RT-qPCR was performed using a CFX Connect (Bio-Rad Laboratories, Hercules, Califorina, USA) with specific TaqMan probes and primers and using Platinum Quantitative PCR SuperMix-UDG w/ROX (Invitrogen, Thermo Fisher Scientific). Moreover, all probes are 5′FAM3′TAMRA-labeled. Sequences for the probes and primers can be found below

RT-qPCR Primer Sequences

| b-actin | b-actin-fw | aga ggg aaa tcg tgc gtg ac |  |  |
| --- | --- | --- | --- | --- |
|  | b-actin-rv | caa tag tga tga cct ggc cgt |  |  |
|  | b-actin-tp | cac tgc cgc atc ctc ttc ctc cc | 5'Fam 3'Tamra | |
|  |  |  |  |  |
| Cldn1 | mCldn1-fw | ccc atc aat gcc agg tat gaa |  |  |
|  | mCldn1-rv | ggt aag agg ttg ttt tcc ggg |  |  |
|  | mCldn1-tp | ctt tac tgg ctg ggc tgc tgc c | 5'Fam 3'Tamra | |
|  |  |  |  |  |
| Ocln | mOcln-fw | aca aga gaa att ttg atg cag gtc t |  |  |
|  | mOcln-rv | cat cag cag cag cca tgt act c |  |  |
|  | mOcln-tp | aag agc tta cag gca gaa cta gac gac gtc aa | 5'Fam 3'Tamra | |
|  |  |  |  |  |
| Rpl13a | mRpl13a-fw | gcg cct caa ggt gtt gga t |  |  |
|  | mRpl13a-rv | ccc agg taa gca aac ttt ctg g |  |  |
|  | mRpl13a-tp | tgg tcc ctg ctg ctc tca agg ttg tt | 5'Fam 3'Tamra | |
|  |  |  |  |  |
| Tjp1 (=ZO-1) | mTJP1-fw | ttc gag aag ctg gat tcc taa gac |  |  |
|  | mTJP1-rv | cag tcc cag cat ctc gtg g |  |  |
|  | mTJP1-tp | cat ctt tgg acc aat agc tga tgt tgc ca | 5'Fam 3'Tamra | |
|  |  |  |  | |
| Cldn3 |  |  |  | |
|  | mCldn3-fw | act acc ggg cct agg aac tgt c |  | |
|  | mCldn3-rv | caa gta gct gca gtg gcc ac |  | |
|  | mCldn3-tp | aag ccg aat gga caa aga aac ctc gc | 5'Fam 3'Tamra | |
|  |  |  |  | |
| Cldn4 |  |  |  | |
|  | mCldn4-fw | cga gca cag ctg gtc cta cc |  | |
|  | mCldn4-rv | aag ggt tcc atg gca gag c |  | |
|  | mCldn4-tp | cat ggt gtg ctg agt gac tga ctg agg g | 5'Fam 3'Tamra | |
|  |  |  |  | |
| Cldn18 |  |  |  | |
|  | mCldn18-fw | atc atc tcc ggc atc tgt gc |  | |
|  | mCldn18-rv | ggt gta cct ggt ctg aac ggt c |  | |
|  | mCldn18-tp | ttt gcc aac atg ctg gtg acc aac tt | 5'Fam 3'Tamra | |

Flow Cytometry configurations

| **Laser Wavelength (nm)** | **Laser Power (mW)** | **Detector** | **Spectral Range (nm)** | **Dichroic LP Filter (nm)** | **Band Pass Filter (nm)** | **Fluorochrome detected** |
| --- | --- | --- | --- | --- | --- | --- |
| 405 (violet) | 50 | V785 | 755-815 | 735 | 785/60 | N/A |
|  |  | V711 | 698.5-723.5 | 685 | 711/25 | Brilliant Violet 711 |
|  |  | V661 | 651-671 | 630 | 661/20 | N/A |
|  |  | V610 | 600-620 | 600 | 610/20 | Brilliant Violet 605 |
|  |  | V525 | 505-535 | 505 | 525/50 | Zombie Aqua |
|  |  | V450 | 425-475 | - | 450/50 | Brilliant Violet 421 |
| 488 (blue) | 50 | B710 | 685-735 | 685 | 710/50 | N/A |
|  |  | B530 | 515-545 | 505 | 530/30 | FITC |
|  |  | SSC | 483-493 | - | 488/10 | Side scatter |
| 561 (yellow-green) | 50 | Y780 | 750-810 | 750 | 780/60 | PE-Cy7 |
|  |  | Y710 | 685-735 | 685 | 710/50 | N/A |
|  |  | Y670 | 655-685 | 635 | 670/30 | N/A |
|  |  | Y610 | 600-620 | 600 | 610/20 | PE-Dazzle594 |
|  |  | Y585 | 578-592 | - | 585/15 | PE |
| 640 (red) | 40 | R780 | 750-810 | 750 | 780/60 | APC-Cy7; FVDeF780 |
|  |  | R730 | 708-750 | 690 | 730/45 | Alexa Fluor 700 |
|  |  | R670 | 663-677 | - | 670/14 | APC-Cy7; FVDeF780 |
